# Thrombotic risk determined by *ABO*, *F8*, and *VWF* variants in a population-based cohort study

**DOI:** 10.1016/j.rpth.2025.102875

**Published:** 2025-04-27

**Authors:** Eric Manderstedt, Christer Halldén, Christina Lind-Halldén, Johan Elf, Peter J. Svensson, Gunnar Engström, Olle Melander, Aris Baras, Luca A. Lotta, Regeneron Genetics Center, Goncalo Abecasis, Aris Baras, Michael Cantor, Giovanni Coppola, Aris Economides, Luca A. Lotta, John D. Overton, Jeffrey G. Reid, Alan Shuldiner, Bengt Zöller

**Affiliations:** 1 Center for Primary Health Care Research, Lund University and Region Skåne, Malmö, Sweden; 2 Department of Bioanalysis, Kristianstad University, Kristianstad, Sweden; 3 Department of Clinical Sciences, Lund University, Skåne University Hospital, Malmö, Sweden; 4 Regeneron Genetics Center, Tarrytown, New York, USA

**Keywords:** ABO blood-group system, factor VIII, molecular epidemiology, venous thromboembolism, von Willebrand factor

## Abstract

**Background:**

Von Willebrand factor (VWF) and coagulation factor VIII (FVIII) plasma levels are associated with increased risk for venous thromboembolism (VTE).

**Objectives:**

This study aimed to determine the thrombotic risk of rare and common variants of 27 genes linked to VWF or FVIII plasma levels in genome-wide association studies.

**Methods:**

Exon sequences of 27 genes linked to plasma levels of VWF or FVIII in genome-wide association studies were analyzed for common and rare variants in 28,794 subjects without VTE (born during 1923-1950, 60% women), who participated in the Malmö Diet and Cancer study (1991-1996), with a follow-up time until 2018. Hazard ratios (HRs) were determined. *P* values were Bonferroni-corrected (*P* value = .05/27 <.0019). Common variants were analyzed individually. Rare qualifying variants (<0.1%) were collapsed.

**Results:**

None of the 27 genes were associated with VTE in the rare variant collapsing analysis. Three common exon variants were significantly associated with VTE: rs8176719 (frameshift) in *ABO* (HR = 1.30; 95% CI, 1.20-1.42; *P* = 3.9 × 10^−10^), rs1800291 (p.Asp1260Glu) in *F8* (HR = 1.29; 95% CI, 1.08-1.55; *P* = .00046 for men; HR = 1.17; 95% CI, 1.06-1.29; *P* = .00019 for women), and rs1063856 (p.Thr789Ala) in *VWF* (HR = 1.10; 95% CI, 1.04-1.17; *P* = .00057). A risk score of these 3 variants was dose-dependently associated with VTE (5 risk alleles): HR = 2.8; 95% CI, 1.7-4.7; and *P* value = .00008. The area under the curve for VTE in receiver operating characteristics for the risk score was similar to FV Leiden (0.55 vs 0.54).

**Conclusion:**

The risk score of 3 common variants in *VWF*, *F8*, and *AB0* genes is associated with VTE risk similar to FV Leiden.

## Essentials

• We exon-sequenced 27 genes linked to the plasma levels of factor VIII and von Willebrand factor (VWF).
• The study was a population-based cohort study of 28,794 middle-aged and older individuals.
• Three common variants in the ABO, F8, and VWF were linked to venous thromboembolism.
• A 3-variant risk score was created that was similar to that for factor V Leiden to predict VTE.

## 1 Introduction

Coagulation factor VIII (FVIII) and its carrier protein von Willebrand factor (VWF) regulate hemostasis and thrombosis [[1], [2], [3], [4], [5]]. Deleterious mutations in the *F8* gene result in the X-linked bleeding disorder hemophilia A [6], whereas mutations in the *VWF* gene that result in deficient or dysfunctional VWF in the plasma lead to the bleeding disorder von Willebrand disease (VWD) [7]. Higher plasma levels of these factors (ie, FVIII and VWF) have been associated with the risk of arterial and venous thrombosis, whereas lower levels are associated with both bleeding disorders and the reduced risk of thrombosis [[1], [2], [3], [4], [5]]. Simioni et al. [8] reported the first thrombophilic rare gain of function mutation in the *F8* gene (designated FVIII Padua) that was associated with markedly elevated FVIII levels and severe thrombophilia in 2 Italian families [8]. In 1969, the ABO blood group was associated with an increased risk of venous thromboembolism (VTE) [9]. The ABO histo-blood group is a major determinant of plasma levels of FVIII and VWF and this association is believed to explain the association between VTE and ABO blood groups [10]. The rs8176719 variant represents an indel in the *ABO* gene, often referred to as either c.261delG or, less commonly, c.260_262insG, and it is a key variant in determining the blood-group type O status [11,12]. The genetics of *ABO* variants and VTE are complex [[13], [14], [15]]. The influence of other variants than rs8176719 on VTE risk has been previously discussed [15]. Besides *ABO* variants, common variations in *VWF* and *F8* genes have been linked to the plasma levels of FVIII and VWF, respectively [[16], [17], [18], [19], [20], [21], [22]].

The importance of *ABO*, *VWF*, and *F8* genes for the development of VTE has been established in genome-wide association studies (GWAS) [[23], [24], [25], [26], [27], [28], [29], [30], [31], [32]]. Several genetic variants in or close (ie, intergenic) to *VWF* (rs1558519, rs216296, rs216311, rs7135039, rs57950734, and rs185699757), *F8* (rs143478537 and rs114209171), and *ABO* (rs1053878, rs495828, rs505922, rs529565, rs582094, rs8176704, rs8176719, rs8176645, rs8176749, rs687289, rs687621, rs2519093, rs579459, rs635634, rs587611953, rs635634, and rs9411395) genes have been associated with VTE in GWAS studies [[23], [24], [25], [26], [27], [28], [29], [30], [31], [32], [33]]. Thus, there is no consensus between studies that are the lead VTE variants in the *ABO*, *F8*, and *VWF* loci [[23], [24], [25], [26], [27], [28], [29], [30], [31], [32], [33]]. Rare variations in the *ABO*, *VWF*, and *F8* genes have not been linked to VTE in whole-exome sequencing (WES) studies [[34], [35], [36], [37], [38]].

FVIII and VWF levels are quantitative traits with high heritability between 60% and 75% [39,40]. A large number of common variants in many loci including the *ABO*, *VWF*, and *F8* genes have been linked to plasma levels of FVIII and its carrier protein VWF in GWAS studies [19,[41], [42], [43], [44], [45], [46], [47]]. Some loci associated with FVIII or VWF plasma levels have also been linked to VTE in GWAS studies [19,[23], [24], [25], [26], [27], [28], [29], [30], [31], [32], [33],[41], [42], [43], [44], [45], [46], [47]]. The loci linked to both FVIII or VWF plasma levels and VTE in GWAS are *ABO*, *VWF*, *F8*, *STAB2*, *STXBP5*, *SCARA5*, *KNG1*, *ST3GAL4*, *HLA*-C, *TAB1*, and *CATSPERB* [19,[23], [24], [25], [26], [27], [28], [29], [30], [31], [32], [33],[41], [42], [43], [44], [45], [46], [47]]. Among the loci associated with FVIII or VWF plasma levels in GWAS studies, so far, only rare variation in the *STAB2* locus has been linked to VTE in WES studies [[34], [35], [36], [37], [38],^48^].

In the present study, we wanted to analyze the exome variation in relation to incident VTE in not only the *ABO*, *F8*, and *VWF* genes but also in other genes that have been linked to plasma levels of FVIII or VWF in GWAS studies [19,[41], [42], [43], [44], [45], [46], [47]]. The coding sequences (CDSs) of 27 genes linked to FVIII or VWF plasma levels in GWAS were analyzed in 28,794 individuals in the large population-based Malmö Diet and Cancer cohort study (MDC) [[48], [49], [50], [51], [52], [53], [54], [55]].

## 2 Methods

### 2.1 Participants

The MDC is a population-based prospective cohort study from the city of Malmö in the south of Sweden [[48], [49], [50], [51], [52], [53], [54], [55]]. Sample characteristics, data collection, and clinical definitions for MDC have been described previously [[48], [49], [50], [51], [52], [53], [54], [55]]. Participants underwent a medical history, physical examination, and laboratory assessment at baseline (1991-1996) [[48], [49], [50], [51], [52], [53], [54], [55]]. Clinical data and DNA were available for 29,387 subjects sampled at baseline. After exclusions of 593 patients with VTE before baseline, a total of 28,794 individuals remained. The study was conducted according to the principles of the Declaration of Helsinki. The Regional Ethics Review Board at Lund University, Lund, Sweden, approved the study (LU 51/90), and all participants provided informed written consent.

### 2.2 Clinical outcomes

One outcome, VTE, was examined. Events were identified through linkage of the 10-digit personal identification number, which is assigned to each resident in Sweden with the Swedish National Patient Registry (SNHDR) and outpatient register. The SNHDR had 100% coverage for inpatients in Malmö during the whole follow-up time and for outpatients from 2001 onward. VTE was defined based on the International Classification of Diseases 7th, 8th, 9th, and 10th revision codes according to Manderstedt et al. [[50], [51], [52], [53], [54], [55]] (Supplementary Table 1). The diagnosis of VTE in the SNHDR has been shown to have an accuracy of 95% [56], whereas the overall validity of the SNHDR is 87% [57]. A quality control of 118 patients with deep venous thrombosis and pulmonary embolism in the MDC was performed [51]. In 106 (90%) of cases, the diagnosis was correct [51]. Patients with VTE at Malmö University Hospital and in Sweden are diagnosed using objective methods [[48], [49], [50], [51], [52], [53], [54], [55], [56],^58^,^59^].

### 2.3 Genetic analysis

WES was performed by Regeneron Genetics Center [60] such that >85% of targeted bases were covered at a read depth of >20×. ANNOtate VARiation was used to aggregate variant annotation, allele frequencies (AFs), and *in silico* predictions of deleteriousness [61]. To control for possible population stratification (ancestry), a principal component analysis (PCA) on common variants was performed and the 2 largest principal components were included in the statistical model. PCA was performed as described elsewhere [62]. The reference genomes were obtained from the 1000 Genomes Project server [62]. The PCA was performed with independent (*R*^2^ measure of linkage disequilibrium [LD] < 0.2) common (minor allele frequency [MAF] ≥ 5%) autosomal biallelic variants that were detected in both the reference genomes and the MDC exomes. To avoid extended LD and high variability regions, such as the major histocompatibility complex, these regions were omitted from the PCA. The principal components were first obtained from the reference genomes and then projected individuals from the MDC onto the principal component space via PLINK2 [62].

### 2.4 Classic thrombophilia

Classic thrombophilia (rs6025 = FV Leiden, rs1799963 = the prothrombin G20210A mutation, protein S deficiency, protein C deficiency, and antithrombin deficiency) was defined as previously described [54]. In short, the hereditary deficiencies of the natural anticoagulants protein S, protein C and antithrombin were defined by pathogenic variants in the Human Gene Mutation Database (HGMD) as described [54,63]. We call them HGMD variants [54].

### 2.5 Gene-collapsing analysis of rare variation

The gene-collapsing approach, in which variants that satisfy specific criteria (qualifying variants) are binned together as equivalent, is an effective approach to identifying rare-variant contributions to disease [[50], [51], [52], [53],^55^]. Exon sequencing identified qualifying variants included in the gene-collapsing analysis of the contribution of rare variants. Qualifying variants (ie, included variants) were defined as loss-of-function (LoF) or nonbenign (PolyPhen-2) missense variants with a MAF of <0.1% [[50], [51], [52], [53],^55^]. Burden tests are not powerful if variants exist with different association directions or many noncausal variants [52,64]. A unified optimal sequence kernel association test was therefore also used to allow different variants to have different directions and magnitude of effects, including no effects [52,64].

### 2.6 Assessing LD

LD for the studied (exonic) and published (exonic, intronic, and intergenic) *ABO*, *VWF*, and *F8* variants was determined using LDlink [65]. LDlink is a web-based collection of bioinformatic modules that query single nucleotide polymorphisms in population groups of interest to generate haplotype tables and interactive plots [65]. Phase 3 haplotype data from the 1000 Genomes Project are referenced for calculating pairwise metrics of LD [65]. The LDmatrix tool was used to create a heatmap matrix of pairwise LD statistics for *ABO*, *VWF*, and *F8* variants among individuals of European descent from Utah and British in England and Scotland [65].

### 2.7 Population frequencies of risk variants

The MAFs in different populations were obtained from the Genome Aggregation Database (gnomAD v4.1.0) [66].

### 2.8 Statistical analysis

The chi-squared test was used to measure differences in allele frequency between study participants with and without VTE. Cox proportional hazards regression was used to examine the association between genotype and incident VTE [54]. Age and sex were included as covariates in the sex- and age-adjusted model, whereas ancestry (PCA), FV Leiden (rs6025), the rs1799963 prothrombin G20210A variant, body mass index (BMI), smoking status, blood pressure (systolic), and high-alcohol consumption (>30 g/d for women, >40 g/d for men) were added in the multivariable model to reduce the statistical noise for these potential general cardiovascular risk factors [[50], [51], [52], [53], [54], [55]]. HGMD variants were further added to a model calculating risk per allele in the created risk score. The fit of the proportional hazards model was checked visually by plotting the incidence rates over time and by calculating Schoenfeld (partial) residuals [54]. Schoenfeld residuals were used as a dependent variable and time as an independent variable to assess the proportional hazards assumption. No violation against proportional hazards assumption was observed. Possible interactions between gene variants and FV Leiden (rs6025), the rs1799963 prothrombin G20210A variant on VTE, were explored by introducing interaction terms in the multivariable models [54]. No interactions were observed. The subjects were categorized according to genotype, and Kaplan–Meier plots were calculated for VTE. For curve comparisons, the log-rank test was used. The area under the receiver operating characteristic curve (AUC) was used as a measure of the overall discriminatory performance of the model as the AUC reflects the probability that the diagnostic test will classify correctly [49]. Logistic regression was used as a first step to create and evaluate a 3-variant risk score that was then used in Cox regression to determine HR, as suggested by Staley et al. [67]. Logistic regression may inflate the effect size especially with single nucleotide polymorphism with great effect size [67]. For prevalent VTE (VTE between 1970 and baseline), the odds ratio (OR) was calculated using logistic regression. For comparison, logistic regression was also conducted to calculate multivariable ORs for incident VTE. Both logistic regression and Cox regression were also performed for recurrent VTE (≥2 incident VTEs during follow-up) compared with those without VTE event. The same adjustments were used in both the logistic regression and Cox regression models. Prevalent VTE cases were excluded for determination of risk for incident and recurrent VTE during follow-up [54]. A sensitivity analysis was performed with exclusion of prevalent cancer and cancer-related VTE. The predictive risk score model was evaluated not only on its discriminatory performance. We also checked the calibration of the model by assessing the agreement between the predicted probability and the observed frequency of outcomes, as described in Staley et al. [67]. R (version 4.0.0 or 4.4.2) was used for all statistical analyses [68]. *P* values (.05) were Bonferroni-corrected according to the number of analyzed genes (*P* values = .05/27 <.0019).

## 3 Results

WES was performed on a total of 28,794 individuals without previous VTE from the MDC cohort, which were available for analysis. Out of these, 2584 (9%) individuals were affected by a VTE (1030 men, 1554 women) event during follow-up until December 31, 2018 (50–55). Mean age at first VTE was 73.7 years (SD, 8.6 years). The sum of the follow-up time was 587,992 years, corresponding to a VTE incidence rate of 4.4 (95% CI, 4.2-4.6) per 1000 person-years [[50], [51], [52], [53], [54], [55]].

### 3.1 Gene-collapsing analysis of rare variants and VTE risk

No significant association was observed in the gene-collapsing analysis using optimal sequence kernel association test for rare variation among the 27 investigated genes after Bonferroni correction (Table 1). Neither did Kaplan–Meier analysis of the collapsed rare variants in any of the 27 genes reveal any significant associations (Supplementary Figures 1-28). The strongest association was with the *STAB2* gene (*P* = .01) as previously reported (Table 1) [50].

**Table 1:** Results of exon sequencing of 27 genes linked to the coagulation factor (FVIII) or VWF plasma levels in GWAS.

Gene	rsID	HR	95% CI	Common *P* value	SKAT-O *P* value
*ABO*^a^^b^	rs8176719	1.30	1.20-1.42	3.9 × 10^−10^^c^	.19
*F8*, females	rs1800291	1.17	1.06-1.29	.00019^c^	.05
*F8*, males	rs1800291	1.29	1.08-1.55	.00046^c^	.42
*VWF*^a^^b^	rs1063856	1.10	1.04-1.17	.00057^c^	.38
*KNG1*^a^	rs1469859	1.10	1.04-1.16	.0019	.07
*GIMAP4*^b^	rs61741802	1.17	1.04-1.32	.0082	.20
*SCARA5*^a^^b^	rs10103504	0.93	0.88-0.98	.015	.82
*CD36*^a^	rs143118835	0.75	0.59-0.96	.022	.39
*HLA*-C^b^	rs1131015	0.96	0.91-1.00	.045	.13
*TAB1*^a^^b^	rs143506704	1.53	1.01-2.33	.047	.23
*STXBP5*^a^^b^	rs34677388	1.16	0.99-1.35	.051	.49
*B3GNT2*^a^	rs148241288	0.71	0.48-1.04	.080	.81
*STAB2*^a^^b^	rs2271637	1.05	0.99-1.11	.080	.01
*TMEM171*^b^	rs637450	0.95	0.89-1.01	.13	.16
*GIMAP7*^b^	rs3735081	0.96	0.91-1.01	.14	1.00
*C2CD4B*^b^	rs8040712	1.06	0.98-1.14	.16	.11
*KIRREL3*^a^^b^	rs948052	0.96	0.91-1.02	.19	.42
*STX2*^b^	rs17564	0.97	0.91-1.02	.22	.56
*TMEM17*^a^	rs11676567	0.97	0.91-1.02	.24	.33
*ST3GAL4*^a^^b^	rs2230279	0.97	0.91-1.03	.26	.28
*F12*^a^	rs17876047	1.08	0.94-1.25	.28	.70
*FCHO2*^a^^b^	rs185435	0.96	0.89-1.05	.36	.03
*CATSPERB*^a^^b^	rs1620238	1.02	0.96-1.09	.51	.39
*ASGR1*^a^	rs55714927	1.02	0.96-1.09	.53	.47
*CLEC4M*^a^^b^	rs2277998	0.98	0.93-1.04	.55	.13
*GRK6*^a^	rs335435	1.01	0.96-1.07	.66	1.00
*PXK*^b^	rs34579268	1.01	0.95-1.07	.79	.20
*RP1*^a^	rs1437782	1.00	0.94-1.05	.88	.42

HRs for VTE with 95% CIs, *P* values for the lead common variant, the SKAT-O test for the rare variant collapsing analysis. All analyses were multivariable and adjusted for age, sex (except sex-stratified *F8* models), BMI, smoking, high-alcohol consumption, 2 first PCA components, rs6025, and rs1799963.

rs1800291 = X-154930010-G-C corresponding to p.Asp1260Glu. No individuals carried the X-154930010-G-A or X-154930010-G-T variants.

BMI, body mass index; HR, hazard ratio; FVIII, factor VIII; GWAS, genome-wide association studies; SKAT, sequence kernel association test; SKAT-O, sequence kernel association-optimal test; VTE, venous thromboembolism; VWF, von Willebrand factor.

a Locus linked in GWAS with FVIII levels.

b Locus linked in GWAS with VWF levels [19,[41], [42], [43], [44], [45], [46], [47]].

c Significant Bonferroni-corrected *P* value = .05/27 <.0019.

### 3.2 Common variants and VTE risk

All coding common variants in the 27 genes were investigated for possible association with VTE (Table 1). Table 1 and Supplementary Figures 1 to 28 present the lead variant in each gene. Three genes *ABO* (rs8176719), *F8*, (rs1800291), and *VWF* (rs1063856) accommodate common variants that were significantly associated with VTE after Bonferroni correction for multiple testing of the 27 genes (Figure 1A-C and Tables 1 and 2). The rs1469859 (*KNG1*) and rs61741802 (*GIMAP4*) variants were almost significant (Table 1). Three alleles, 1 common and 2 rare, are described for the rs1800291 variants in gnomAD [66]. However, all individuals in the study population carried the X-154930010-G-C (Table 1) variant corresponding to p.Asp1260Glu. Below presented is a detailed description of variants in the 3 genes (*ABO*, *F8*, and *VWF*) harboring the 3 significant common lead variants associated with VTE.

**Figure 1:**
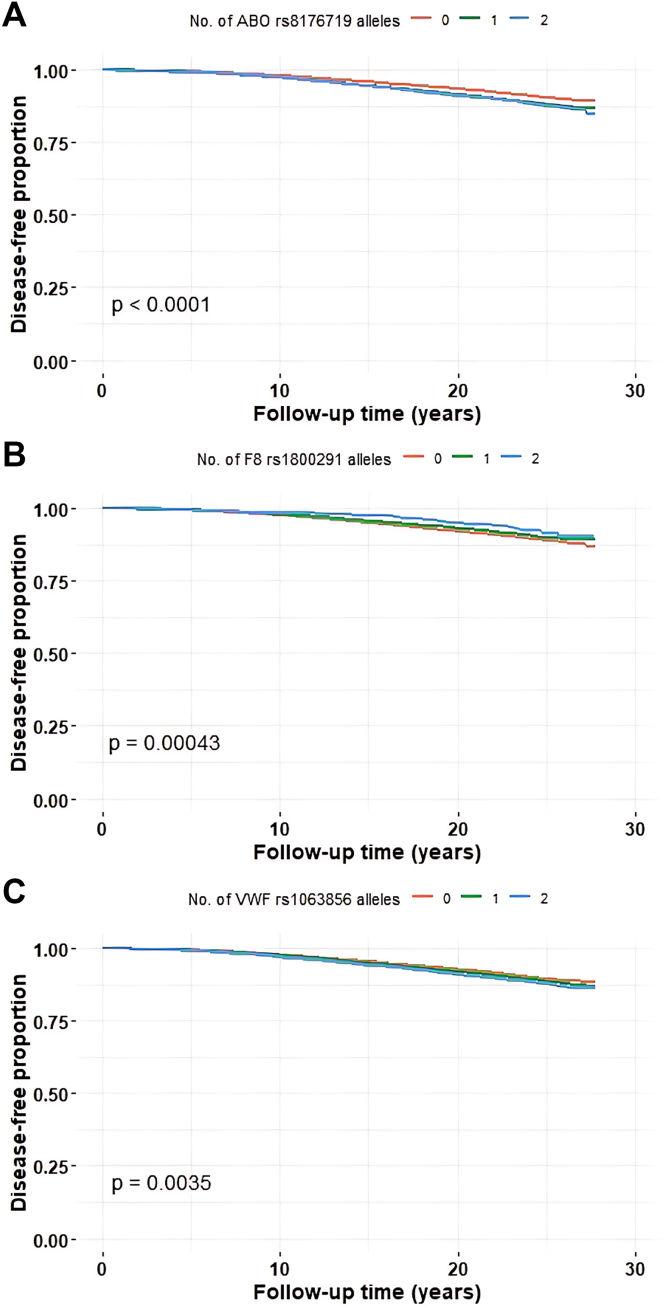
Kaplan–Meier curves for the 3 lead variants associated with VTE risk: (A) rs8176719 in *ABO*, (B) rs1800291 in *F8* (for females), and (C) rs1063856 in *VWF*. VTE, venous thromboembolism; VWF, von Willebrand factor.

**Table 2:** HRs for incident VTE for rs8176719, rs1800291, and rs1063856 adjusted for either age and sex (except sex-stratified *F8* models) or multivariable HRs adjusted for age, sex, BMI, smoking, high alcohol consumption, 2 first PCA components, rs6025, and rs1799963.

Gene variants	Participants with no VTE	VTE	Crude IR	Crude IRR	Age- and sex-adjusted HR *P* value	Multivariable HR *P* value
*ABO*						
Reference no variant	9926	822	3.7 (3.5-4.0)	1	1	1
≥1 AB0 rs8176719	16,284	1762	4.7 (4.5-5.0)	1.3 (1.2-1.4)	1.2 (1.1-1.2), *P* = 4 × 10^−9^	1.2 (1.1-1.3), *P* = 2 × 10^−9^
*F8*						
Reference females no variant	491	34	3.1 (2.1-4.3)	1	1	1
per allele rs1800291 females	15,327	1520	4.3 (4.1-4.5)	1.3 (0.9-1.8)	1.2 (1.1-1.3), *P* = 2 × 10^−3^	1.2 (1.1-1.3), *P* = 2 × 10^−3^
Reference males no variant	1764	140	3.8 (3.2-4.4)	1	1	1
= 1 rs1800291 allele males	8628	890	4.8 (4.5-5.2)	1.3 (1.1-1.5)	1.3(1.1-1.6), *P* = 3 × 10^−3^	1.3 (1.1-1.5), *P* = 5 × 10^−3^
*VWF*						
Reference no variant	11,075	1016	4.1 (3.9-4.4)	1	1	1
per allele rs1063856	15,135	1568	4.5 (4.3-4.8)	1.1 (1.0-1.2)	1.1 (1.0-1.2), *P* = 7 × 10^−4^	1.1 (1.0-1.2), *P* = 5 × 10^−4^

IRs and IRRs are also presented. Prevalent cases of VTE were excluded. rs1800291 = X-154930010-G-C corresponding to p.Asp1260Glu. No individuals carried the X-154930010-G-A or X-154930010-G-T variants.

IR, incidence rate; IRR, incidence rate ratio; *P* = *P* value.

### 3.3 Common variation in the *ABO* gene

Variant allele frequencies (VAFs) and CDS positions for all detected *ABO* variants are presented in Supplementary Figure 1. There were 7 LoF, 66 missense and 36 synonymous variants. A total of 31 variants had a VAF of >0.1% and 25 had a VAF of >1%. The causal frameshift variant for the blood group O, rs8176719, showed the largest frequency difference between individuals with or without VTE (chi-squared test, *P* value = 1 × 10^−8^). A number of additional variants had chi-squared test *P* values of <.05: rs8176720 (p.Thr98=, *P* value = .011), rs512770 (p.Pro74Ser, *P* = .0021), and rs8176745 (p.Pro256=, *P* value = .0047). However, after adjustment for the lead ABO variant rs8176719 (Table 1), no variant was significantly associated with VTE (Supplementary Table 2). The Kaplan–Meier analysis for rs8176719 is shown in Figure 1A and Supplementary Figure 1.

### 3.4 Common variation in the *F8* gene

VAFs and CDS positions for all detected *F8* variants are presented separately for women and men (Supplementary Figures 2 and 3). There were 3 LoF, 127 missense, 52 synonymous, and 11 untranslated region variants. Five variants showed a VAF of >0.1%. Two had VAFs of >1%: rs1800292 (p.Ser1288=) and rs1800291 (p.Asp1260Glu, X-154930010-G-C). Only the lead variant rs1800291 was associated with VTE in both men and women (chi-squared test, *P* value = 2 × 10^−6^; Table 1). No association with VTE was observed for the rs1800292 variant (*P* value = .63) or for any other variant after adjustment for rs1800291. The minor allele of rs1800291 had a protective effect in relation to VTE. The Kaplan–Meier analysis for rs1800291 is shown in Figure 1B and Supplementary Figures 2 and 3.

### 3.5 Common variation in the *VWF* gene

VAFs and CDS positions for all detected *VWF* variants and the differences in frequency between individuals with or without VTE are shown in Supplementary Figure 4. There were 14 LoFs, 338 missense, 195 synonymous, and 2 untranslated region variants. A total of 53 variants had a VAF of >0.1%, whereas 31 variants had a VAF of >1%. The 2 variants showing the largest frequency differences, rs1063856 (p.Thr789Ala, chi-squared test *P* value = 9 × 10^−4^) and rs1063857 (p.Tyr795=), are located close to each other and in complete LD (*R*^2^ = 1.0) with each other. The only other variant showing a significant frequency difference between individuals with or without VTE was rs1800379 (p.Tyr516=, *P* value = .022). The Kaplan–Meier analysis for rs1063856/rs1063857 is shown in Figure 1C and Supplementary Figure 4. We use the missense variant rs1063856 though it is completely interchangeable with synonymous variant rs1063857 (Table 1).

### 3.6 Combined effect of VTE risk alleles in *ABO*, *F8*, and *VWF*

**Figure 2:**
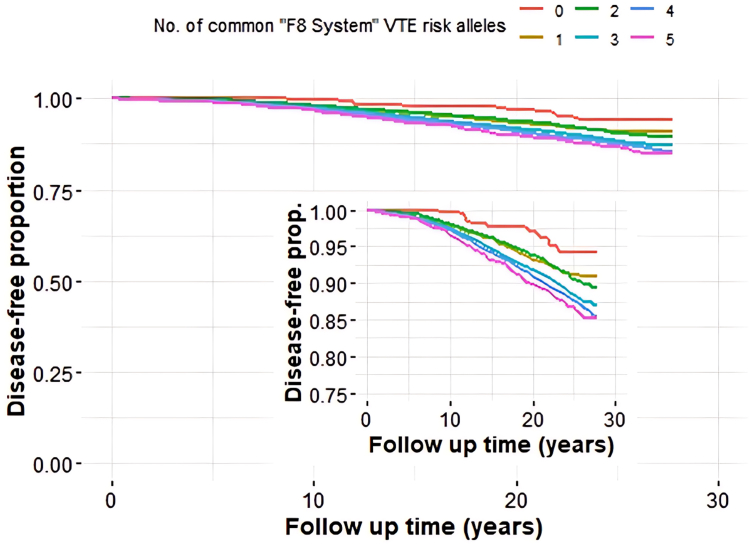
Kaplan–Meier curves for the number of risk alleles in the 3-variant risk score (rs8176719, rs1800291, and rs1063856). The following model is used: risk is increased for one or more of the insertion rs8176719 in *ABO*, for every major allele of rs1800291 in *F8* (hemizygote men are modeled as carrying 2 risk alleles in *F8*), or for each minor allele of rs1063856 in *VWF*. A broken *y*-axis is inlaid for clarity. VTE, venous thromboembolism; VWF, von Willebrand factor.

A risk score was created by counting the number of alleles of the 3 significant independent variants: rs8176719 in *AB0*, rs1800291 in *F8*, and rs1063856 in *VWF* (Table 1). In *ABO*, the risk allele is the insertion allele of rs8176719 that results in a functional protein. Supplementary Figure 29 shows that both heterozygosity and homozygosity for this allele result in similar ORs of 1.2, whereas the total absence of this allele results in an OR of 0.8. Thus, the insertion allele has a dominant effect and is therefore coded as either 0 or 1. For both *F8* and *VWF*, the ORs increased with the number of risk alleles (the major allele of rs1800291 in *F8* and the minor allele of rs1063856 in *VWF*; Supplementary Figure 29). We found a strong association between the number of risk alleles and the incident risk of VTE, with higher VTE incidence with an increasing number of alleles (Figure 2, Table 3, and Supplementary Table 3). The effect size of the 3-variant risk score is within the range of classic thrombophilia in this cohort (Table 3). Supplementary Figure 30 shows the proportions of individuals in the total population with different numbers of risk alleles. Since all 3 variants have high population frequencies, individuals with all combinations of risk alleles could be found and showed ORs as could be expected from the data on individual variants (Supplementary Figure 31).

**Table 3:** HRs for the 3 variant risk score (rs8176719, rs1800291, rs1063856) for incident VTE adjusted for either age and sex or multivariable HRs adjusted for age, sex, BMI, smoking, high alcohol consumption, 2 first PCA components, rs6025, and rs1799963.

*N* alleles	Participants with no VTE	VTE	Age- and sex-adjusted HR (95% CI)	*P* value	Multivariable HR (95% CI)	*P* value
Females (not adjusted for sex)						
0 Alleles	70	3	1	Reference	1	Reference
1 Allele	908	73	2.0 (0.6-6.2)	.25	2.2 (0.7-7.1)	.17
2 Alleles	3834	295	1.9 (0.6-5.8)	.28	2.2 (0.7-6.8)	.18
3 Alleles	6232	649	2.5 (0.8-7.9)	.11	2.9 (0.9-9.1)	.06
4 Alleles	3938	432	2.7 (0.9-8.3)	.09	3.1 (1.0-9.7)	.05
5 Alleles	836	102	3.0 (0.9-9.3)	.06	3.5 (1.1-11.1)	.03
Males (not adjusted for sex)						
0 Alleles	281	13	1	Reference	1	Reference
1 Allele	2193	165	1.7 (1.0-3.0)	.07	1.6 (0.9-2.9)	.09
2 Alleles	4304	429	2.3 (1.3-3.9)	.004	2.2 (1.3-3.8)	.005
3 Alleles	2945	338	2.6 (1.5-4.5)	.0008	2.5 (1.4-4.3)	.001
4 Alleles	669	85	2.8 (1.6-5.0)	.0006	2.6 (1.5-4.7)	.001
Both sexes (hemizygosity coded as homozygosity)						
0 Alleles	351	16	1	Reference	1	Reference
1 Allele	1656	127	1.5 (1.0-3.0)	.03	1.7 (1.0-2.9)	.04
2 Alleles	5863	465	1.8 (1.1-3.0)	.02	1.8 (1.1-3.0)	.02
3 Alleles	10,103	1033	2.4 (1.4-3.9)	.001	2.3 (1.4-3.8)	.0007
4 Alleles	6732	756	2.6 (1.6-4.2)	.0002	2.6 (1.6-4.2)	.0002
5 Alleles	1505	187	2.8 (1.7-4.7)	.00008	2.8 (1.7-4.7)	.00008
Assuming a multiplicative effect per risk allele (hemizygosity coded as homozygosity) for both sexes’ multivariable model + HGMD (SERPINC1, PROC, and PROS1 variants)						
Risk score per allele			1.2 (1.1-1.2)	3 × 10^−14^	1.2 (1.1-1.2)	2 × 10^−14^
rs6025 per allele^a^			1.8 (1.6-2.0)	5 × 10^−37^	1.8 (1.6-2.0)	5 × 10^−37^
rs1799963 per allele^a^			1.6 (1.3-2.0)	2 × 10^−5^	1.6 (1.3-2.0)	2 × 10^−5^
HGMD per allele^a^			1.6 (1.3-1.9)	1 × 10^−6^	1.6 (1.3-1.9)	5 × 10^−7^

BMI, body mass index; HGMD, Human Gene Mutation Database; PCA, principal component analysis; VTE, venous thromboembolism.

a rs6025, 25,562 individuals with no allele (2106 had VTE), 3129 heterozygotes (450 had VTE), 103 homozygotes (28 had VTE); rs1799963, 28,268 individuals with no allele (2511 had VTE), 524 heterozygotes (72 had VTE), 2 homozygotes (1 had VTE); and HGMD variants, 27,920 individuals with no variant (2465 had VTE), 868 individuals with 1 variant (117 had VTE), and 6 individuals with 2 variants 6 (2 had VTE).

The calculated AUC for the 3-variant risk score for VTE was 0.55 (95% CI, 0.54-0.56), similar to the AUC of rs6025 (0.54; 95% CI, 0.53-0.55) but higher than the AUCs for rs1799963 (0.51; 95% CI, 0.50-0.51) or pathogenic HGMD variants in *SERPINC1*, *PROS1*, or *PROC* genes (0.51; 95% CI, 0.50-0.51; Table 4). In a model with the inclusion of both genetic and other risk factors, the AUC for VTE was 0.62 (95% CI, 0.61-0.63; Table 4).

**Table 4:** ROC with calculation of AUC for the 3-variant risk score (rs8176719, rs1800291, and rs1063856) and other potential predictors for VTE.

Variable name	AUC	Lower 95% CI	Upper 95% CI
Three-variant risk score	0.55	0.53	0.56
rs6025 (Factor V Leiden)	0.54	0.53	0.55
rs1799963 (prothrombin G20210A)	0.50	0.50	0.51
HGMD (*PROS1*, *PROC*, *SERPINC1*)	0.51	0.50	0.51
BMI	0.56	0.55	0.57
Age + sex + PCA	0.56	0.55	0.58
Systolic BP	0.52	0.51	0.53
All factors above	0.62	0.61	0.63

AUC, area under the curve; BMI, body mass index; BP, blood pressure; HGMD variant, pathogenic variant in *PROS1*, *PROC*, or *SERPINC1* according to Manderstedt et al. [54]; PCA, principal component analysis; ROC, receiver operating characteristic; VTE, venous thromboembolism.

### 3.7 Association with prevalent VTE and recurrent VTE

For comparison between prevalent and incident VTE, OR was also determined for incident VTE. Assuming a multiplicative effect per risk allele, the adjusted multivariable OR for incident VTE was 1.2 (95% CI, 1.1-1.2) with a *P* value of 6 × 10^−14^ (not in tables). This is similar to the calculated HR per allele (1.2; 95% CI, 1.1-1.2) displayed in Table 3 with a *P* value of 2 × 10^−14^. A similar multivariable OR was observed for prevalent VTE cases (*n* = 593) per risk allele: 1.1 (95% CI, 1.0-1.3) with a *P* value of.009. Calculating multivariable OR for cases with recurrent VTE (*n* = 1491), the multivariable OR per risk allele was 1.2 (95% CI, 1.2-1.3) with a *P* value of 3 × 10^−14^, which was identical to the multivariable HR for individuals with recurrent VTE of 1.21 (95% CI, 1.2-1.3, *P* value = 8 × 10^−15^).

### 3.8 LD between variants in *ABO*, *F8*, and *VWF* using LDlink

The rs8176719 (*ABO*) has previously been identified in a GWAS study [23]. In Supplementary Figure 32, a heatmap is displayed with other ABO gene variants. A strong LD was observed with several previous variants associated with VTE in GWAS studies (Supplementary Table 4) [[23], [24], [25], [26], [27], [28], [29], [30], [31], [32], [33]]. The *F8* rs1800291 is not in LDlink but has previously been shown to be in LD to a *F8* variant rs114209171 linked to VTE in a GWAS study (*R*^2^ = 0.28) [26]. In LDlink, the rs114209171 variant is also linked to another GWAS VTE-linked *F8* variant rs143478537 (*R*^2^ = 0.2563). The *VWF* variant rs1063856 is in strong LD with GWAS-linked VTE variants rs1558519 and rs7135039 (Supplementary Figure 33 and Supplementary Table 5) [28,30,31].

### 3.9 Population frequencies of rs8176719 (*ABO*), rs1800291 (*F8*), and rs1063856 (*VWF*)

All 3 variants (rs8176719, rs1800291, and rs1063856) are common with MAFs of 5% or more among all different populations represented in gnomAD v4.1.0 (Supplementary Table 6) [66].

### 3.10 Additional analysis

A sensitivity analysis was performed with the exclusion of prevalent cancer and cancer-related VTE. However, the 3-variant risk score was still associated with VTE. In study participants without cancer-related VTE, the multivariable HR for incident VTE was 1.18 (95% CI, 1.1-1.3, *P* value = 8 × 10^−9^) per risk allele.

Extensive calibration of the model was performed. We show one of a large number of tested models because our model was robust with only minor differences (Supplementary Figure 34). Calibration was done by performing the training of the model on 70% of the population (*n* = 20,571 individuals) and tested in the remainder (*n* = 8816). The calibration of the model showed an intercept of −0.03 (95% CI, −0.10 to 0.03) and a slope of 0.95 (95% CI, 0.82-1.09). Building a model using only incident cases tended to create a situation where the observed proportion of VTE cases was lower than the predicted probability (not shown) when the predicted probability was high. Including the prevalent VTE cases alleviated this, possibly due to that many high-risk individuals already are affected by prevalent VTE before the study baseline.

## 4 Discussion

The present study design, which includes only genes significantly linked to coagulation FVIII and VWF plasma levels in GWAS studies [[40], [41], [42], [43], [44], [45], [46]], gives a functional background to our results as coagulation FVIII and VWF plasma levels are linked to VTE risk [[1], [2], [3], [4], [5],^8^]. We found 3 independent *ABO*, *F8*, and *VWF* variants that are all likely functional [[9], [10], [11], [12], [13], [14], [15], [16], [17], [18], [19], [20], [21], [22]] to be significantly associated with VTE and created a risk score linked to incident VTE risk in a dose-dependent way. The risk score was also associated with prevalent VTE and recurrent VTE. Thus, a simple score consisting of 3 variants in the *ABO*, *F8*, and *VWF* genes that are linked to FVIII and VWF plasma levels is a predictor for VTE in a Swedish population of middle-aged and older individuals. The present 3-variant risk score may be of greater value than the rs6025 and rs1799963 variants because they mainly affect Caucasians [33,54,66], whereas the 3 variants in the present study are common among all ethnic groups in gnomAD (Supplementary Table 6). The results suggest that genetically determined FVIII and VWF plasma levels are important determinants of the VTE risk in the general population. The dose-dependent increased risk for VTE according to the number of alleles was not linked to any special combination of the 3 variants (Supplementary Figure 31). Most importantly, the created 3-variant risk score is just as important as rs6025 in the present study population (Tables 3 and 4) [54]. The 3-variant risk score also had a higher AUC than rs1799963 and genetic defects in the *PROS1*, *PROC*, and *SERPINC1* genes (Table 4) [54]. The HR and OR are also within the range of these 5 classic thrombophilias in the present population-based cohort of middle-aged and older individuals [54]. The prevalence of many complex diseases such as VTE increases steeply with age, whereas often the familial risk declines [69]. For instance, the heritability and familial risk for VTE is age-dependent in Swedish families [70,71]. Moreover, the OR for deep venous thrombosis among FV Leiden carriers is higher for younger than older individuals [72]. This may explain the low genotype risk associated with classical thrombophilia reported in the present population-based study of middle-aged and older individuals [54]. We therefore believe that the reported risk score will be stronger among younger individuals. Of interest is that this genetic score for FVIII levels affects the first step in the common coagulation pathway, whereas rs6025 affects the second step, and rs1799963 affects the last step in the common coagulation pathway. Antithrombin affects all 3 steps, and the protein C anticoagulant system affects the first 2 steps of the common coagulation pathway. Disturbances in the regulation of the common coagulation pathway are thus crucial for classic thrombophilia and hypercoagulability.

The present study could not identify any significant association after Bonferroni correction between rare variants and VTE in the gene-collapsing analysis of the 27 associated with plasma levels of coagulation FVIII and VWF (Table 1) [19,[41], [42], [43], [44], [45], [46], [47]]. These results confirm previous large WES studies that could not find associations between rare variants and VTE in the gene-collapsing analysis of the 27 genes associated with the plasma levels of coagulation FVIII and VWF [[35], [36], [37], [38]]. The strongest association in the gene-collapsing analysis was observed for the *STAB2* gene (*P* = .01; Table 1) [50]. Desch et al. [34] has previously found an association with VTE and rare variation in the *STAB2* gene [34].

In the present study of the coding regions, the thrombotic risk of the *ABO* locus was best reflected by the causal frameshift variant for blood group O, rs8176719 [11,12]. No other independent signal could be identified. The rs8176719 has previously been linked to VTE in the GWAS study, but the variant is also in strong LD with several VTE-linked variants in GWAS studies: rs687621 (*R*^2^ = 0.868), rs687289 (*R*^2^ = 0.868), rs582094 (*R*^2^ = 0.857), rs8176645 (*R*^2^ = 0.977), rs505922 (*R*^2^ = 0.869), and rs529565 (*R*^2^ = 0.857) [[23], [24], [25], [26], [27], [28], [29], [30], [31], [32], [33]] (Supplementary Table 4). Thus, rs8176719 is a robustly confirmed VTE GWAS variant. The *F8* rs1800291 is not present in LDlink but has previously been shown to be in LD to the GWAS-positive *F8* gene variant rs114209171 (*R*^2^ = 0.28) [26]. The rs1800291 (p.Asp1260Glu) in the *F8* gene has previously also been reported to be associated with the plasma levels of FVIII [22,73]. It was not possible to link this variant with the upstream *F8* variant rs143478537 that has been linked to VTE in GWAS [28] in LDlink. The *VWF* lead variant rs1063856 (p.Thr789Ala) is in strong LD with 2 variants associated with VTE in GWAS: rs1558519 (*R*^2^ = 1.0) and rs7135039 (*R*^2^ = 0.924) [[23], [24], [25], [26], [27], [28], [29], [30], [31], [32], [33]]. Thus, the rs1063856 variant is also an indirectly GWAS-confirmed VTE variant. Moreover, the rs1063856 variant has been linked to plasma levels of VWF [[16], [17], [18], [19], [20], [21], [22]]. The missense rs1063856 (p.Thr789Ala) variant could be the functional culprit variant for the published VTE associations in GWAS studies for rs1558519 and rs7135039.

Thus, exon sequencing has identified 3 functional variants in the *ABO* (frameshift mutation causing blood group O), *VWF* (missense p.Thr789Ala), and *F8* (missense p.Asp1260Glu) to be associated with VTE in the present study. They have previously been identified in GWAS studies [[23], [24], [25], [26], [27], [28], [29], [30], [31], [32]] and have also been associated with the plasma levels of VWF or FVIII [[16], [17], [18], [19], [20], [21], [22],^73^]. The present strategy to exon-sequence GWAS-linked genes seems to be a powerful option to uncover the functional mutations associated with both plasma levels of coagulation FVIII and VWF and also with VTE.

### 4.1 Strengths and limitations

The study cohort included many subjects and events during a long follow-up period [[48], [49], [50], [51], [52], [53], [54], [55]]. The cardiovascular outcomes were obtained from national registers covering all of Sweden [[48], [49], [50], [51], [52], [53], [54], [55]]. Validation studies of cases obtained from the Swedish hospital discharge register have shown a 90% to 95% validity of VTE [51,56]. A previous study from the Malmö University Hospital between 1998 and 2006 has shown that virtually all VTE patients are diagnosed with an objective method such as phlebography, ultrasound, or computer tomography [58], which is in line with Swedish recommendations that all VTE events should be objectively verified [59].

A limitation is the lack of confirmation in another cohort. However, a strength is the internal replication of the 3-variant risk score with prevalent and recurrent VTE and the robustness of our model observed when performing calibration. Exclusion of prevalent cancer and cancer-related VTE did not change the results. Another strength is that the lead variants included in the risk score are either identified in GWAS studies of VTE or linked to variants associated with VTE in GWAS studies [[23], [24], [25], [26], [27], [28], [29], [30], [31], [32], [33]]. Although inherited factors are significant risk factors for VTE in the present age group [[48], [49], [50], [51], [52], [53], [54], [55]], inherited factors are relatively more important at younger ages [54,[69], [70], [71], [72]]. It is possible that the risk score will be more strongly associated with VTE among younger patients. It will be important to verify the present results in studies with information on circumstantial risk factors. Notable is that the AUC was like FV Leiden and higher than for other classic thrombophilias in the present study population of middle-aged and older individuals (Table 4) [54]. This suggests the 3-variant risk score to be of importance because the prevalence of FV Leiden is quite high in Sweden [54,72]. The MDC population has only 12% admixture from foreign-born individuals. Among foreign-born individuals, only 1% were non-European [54]. We have no information on ethnicity or race. However, unlike FV Leiden, which is only present in people of Caucasian origin, all 3 variants in the risk score (rs8176719, rs1063856, and rs1800291) are common in all populations in gnomAD independent of ethnic origin (Supplementary Table 6) [66]. The present risk score may therefore turn out to be clinically useful in many ethnic populations and not only among Caucasians. For instance, future studies need to test the value to add this risk score to thrombophilia testing but also as a risk marker in different clinical settings.

A strength but also a limitation of the study design is that we included only genes linked to FVIII and VWF plasma levels in GWAS studies [19,[41], [42], [43], [44], [45], [46], [47]]. Other not GWAS-linked genes involved in the clearance of VWF such as *LPR1*, *ASGR2*, *SIGLEC5*, and *LDLR* are interesting candidate genes for further studies. Still, several included GWAS-linked genes are involved in VWF clearance: *STAB2*, *SCARA5*, *ASGR1*, and *CLEC4M.*

The high VTE incidence rate of 4.4 (95% CI, 4.2–4.6) per 1000 person-years confirms a study from Gothenburg in Sweden with a high VTE incidence of 387 per 100,000 observation-years [74]. Another limitation is the lack of information on anticoagulant and aspirin treatment, but we adjusted for potential cardiovascular risk factors.

In conclusion, the present study suggests that genetic variation in genes linked to coagulation FVIII and VWF plasma levels is an important determinant for VTE risk similar to FV Leiden. Exon sequencing of 27 candidate genes linked to FVIII and VWF plasma levels led to the identification of a simple risk score consisting of 3 independent likely functional variants in the *ABO*, *F8*, and *VWF* genes that is a predictor for VTE. Unlike rs6025 and rs1799993, the risk variants are common in all ethnic populations in gnomAD.

## Appendix

Regeneron Genetics Center banner authors/contributors (listed in alphabetical order) and their contributions.

RGC Management and Leadership Team: Goncalo Abecasis, Aris Baras, Michael Cantor, Giovanni Coppola, Aris Economides, Luca A. Lotta, John D. Overton, Jeffrey G. Reid, and Alan Shuldiner. All authors contributed to securing funding, study design, and oversight and reviewed the final version of the manuscript.

Sequencing and Laboratory Operations: Christina Beechert, Caitlin Forsythe, Erin D. Fuller, Zhenhua Gu, Michael Lattari, Alexander Lopez, John D. Overton, Thomas D. Schleicher, Maria Sotiropoulos Padilla, Louis Widom, Sarah E. Wolf, Manasi Pradhan, Kia Manoochehri, and Ricardo H. Ulloa. C.B., C.F., A.L., and J.D.O. performed and are responsible for sample genotyping. C.B., C.F., E.D.F., M.L., M.S.P., L.W., S.E.W., A.L., and J.D.O. performed and are responsible for exome sequencing. T.D.S., Z.G., A.L., and J.D.O. conceived and are responsible for laboratory automation. M.P., K.M., R.U., and J.D.O. are responsible for sample tracking and the library information management system.

Genome Informatics: Xiaodong Bai, Suganthi Balasubramanian, Andrew Blumenfeld, Boris Boutkov, Gisu Eom, Lukas Habegger, Alicia Hawes, Shareef Khalid, Olga Krasheninina, Rouel Lanche, Adam J. Mansfield, Evan K. Maxwell, Mrunali Nafde, Sean O’Keeffe, Max Orelus, Razvan Panea, Tommy Polanco, Ayesha Rasool, Jeffrey G. Reid, William Salerno, and Jeffrey C. Staples. X.B., A.H., O.K., A.M., S.O., R.P., T.P., A.R., W.S., and J.G.R. performed and are responsible for the computing logistics, analysis, and infrastructure needed to produce exome and genotype data. G.E., M.O., M.N., and J.G.R. provided computing infrastructure development and operational support. S.B., S.K., and J.G.R. provide variant and gene annotations and their functional interpretation of variants. E.M., J.S., R.L., B.B., A.B., L.H., and J.G.R. conceived and are responsible for creating, developing, and deploying analysis platforms and computational methods for analyzing genomic data.

Research Program Management: Marcus B. Jones, Jason Mighty, and Lyndon J. Mitnaul. All authors contributed to the management and coordination of all research activities, planning, and execution and to the review process for the final version of the manuscript.
