# Supplementary material for: Thrombotic risk determined by ABO, F8, and VWF variants in a population-based cohort study
Source: Res Pract Thromb Haemost. 2025 Apr 27;9(4):102875. doi: 10.1016/j.rpth.2025.102875 (PMC12145703; doi:10.1016/j.rpth.2025.102875)

**Supplementary Tables 1-6 and Supplementary figures 1-34.**

| **Supplementary Table 1.** International Classification of Diseases (ICD) 7th, 8th, 9th and 10th revisions codes used for defining venous thromboembolism (VTE). ICD-9 and ICD-10 codes used for follow-up period and ICD-7, ICD-8, and ICD-9 codes used for VTE before baseline. Deep venous thrombosis (DVT) of the legs is marked with * and Pulmonary embolism (PE) with †. | | | |
| --- | --- | --- | --- |
| ICD-10 | ICD-9 | ICD-8 | ICD-7 |
| I26† | 325 | 321 | 33440 |
| I636 | 415B† | 450† | 33450 |
| I676 | 416W† | 451* | 463* |
| I80* | 437G | 452 | 464 |
| I81 | 451* | 453 | 465† |
| I82 | 452 | 671* | 466 |
| O222 | 453 | 6739† | 58300 |
| O223* | 639G† |  | 682* |
| O225 | 671C |  | 684† |
| O229 | 671D* |  |  |
| O082† | 671E* |  |  |
| O870 | 671F |  |  |
| O871* | 671X |  |  |
| O873 | 673C† |  |  |
| O879 |  |  |  |
| O882† |  |  |  |
| *Deep venous thrombosis (DVT) of the legs: excluded I800, 451A and 67100.  †Pulmonary embolism (PE). | | | |

| **Supplementary Table 2**. Hazard ratios (HRs) with 95% confidence intervals (CI) with p-values for ABO variants and incident venous thromboembolism (VTE) adjusted for either age and sex, or multivariable HRs adjusted for age, sex, body mass index (BMI), smoking, high alcohol consumption, two first PCA components, rs6025, and rs1799963. Models without and with adjustment for the ABO lead variant rs8176719. | | | | | | |
| --- | --- | --- | --- | --- | --- | --- |
| *ABO* | Participants without VTE | VTE cases | IR (95% CI) | Crude IRR (95%CI) | Age and sex adjusted HR (95%CI) | Multivariable HR  (95%CI) |
| **rs8176719** | | | | | | |
| Reference = 0 allele | 9926 | 822 | 3.7 (3.5-4.0) | 1 | 1 | 1 |
| ≥one allele | 16284 | 1762 | 4.7 (4.5-5.0) | 1.3 (1.2-1.4) | 1.2 (1.1-1.2)  p-value 4e-9 | 1.2 (1.1-1.3)  p-value 2e-9 |
| **rs512770** | | | | | | |
| Reference = 0 allele | 8817 | 804 | 4.6 (4.3-4.8) | 1 | 1 | 1 |
| ≥one allele | 17393 | 1780 | 4.2 (3.9-4.5) | 0.9 (0.8-1.0) | 0.9 (0.8-1.0) 3e-3 | 0.9 (0.8-1.0)  p-value 6e-3 |
| ≥one allele adjusted for rs8176719 | | | | | | 1. (0.9-1.0)   p-value 0.26 |
| **rs8176720** | | | | | | |
| Reference = 0 allele | 11712 | 1377 | 4.6 (4.3-4.9) | 1 | 1 | 1 |
| ≥one allele | 14498 | 1207 | 4.3 (4.1-4.6) | 09 (0.9-1.0) | 09 (0.9-1.0) 2e-2 | 0.9 (0.9-1.0)  p-value 2e-2 |
| ≥one allele adjusted for rs8176719 | | | | | | 1.0 (0.9-1.0)  p-value 0.10 |
| **rs8176745** | | | | | | |
| Reference = 0 allele | 15477 | 1582 | 4.3 (4.0-4.6) | 1 | 1 | 1 |
| ≥one allele | 10733 | 1002 | 4.6 (4.3-4.8) | 09 (0.9-1.0) | 09 (0.9-1.0)  p-value 4e-3 | 09 (0.9-1.0)  p-value 4e-3 |
| ≥one allele adjusted for rs8176719 | | | | | | 1. (0.9-1.1) 2. p-value 0.73 |
| Abbreviations: CI, confidence interval; IR, incidence rate; IRR, incidence rate ratio; SD, standard deviation. Note: Incidence rates (IRs) and incidence rate ratios (IRRs) are also presented. Prevalent cases of VTE were excluded. | | | | | | |

| **Supplemental Table 3.** Hazard ratios (HRs) for the three variant risk score (rs8176719, rs1800291, rs1063856) for incident venous thromboembolism (VTE) adjusted for either age and *sex, or multivariable HRs adjusted for age, *sex, body mass index (BMI), smoking, high alcohol consumption, two first PCA components, rs6025, and rs1799963. With three risk alleles as reference for calculations. | | | | | | |
| --- | --- | --- | --- | --- | --- | --- |
| N alleles | Participants with no VTE | VTE | Age- and sex-adjusted HR (95%CI) | p-value | Multivariable HR (95%CI) | p-value |
| Both sexes (hemizygosity coded as homozygosity) | | | | | | |
| 0 alleles | 351 | 16 | 0.4 (0.3-0.7) | 0.0007 | 0.4 (0.3-0.7) | 0.0007 |
| 1 alleles | 1656 | 127 | 0.8 (0.6-0.9) | 0.002 | 0.7 (0.6-0.9) | 0.002 |
| 2 alleles | 5863 | 465 | 0.8 (0.7-0.9) | 0.000005 | 0.8 (0.7-0.9) | 0.000007 |
| 3 alleles | 10103 | 1033 | 1 | Reference | 1 | reference |
| 4 alleles | 6732 | 756 | 1.1 (1.0-1.2) | 0.1 | 1.1 (1.0-1.2) | 0.1 |
| 5 alleles | 1505 | 187 | 1.2 (1.0-1.4) | 0.03 | 1.2 (1.0-1.4) | 0.03 |

| **Supplementary Table 4**. Linkage disequilibrium (LD) expressed as R^2^ for ABO variants in relation to the lead ABO variant **rs8176719** determined using Ldlink [66]. The lead variant **rs8176719 (frameshift)** is displayed in bold. | | |
| --- | --- | --- |
| **ABO variants** | **GWAS reference** | **R^2^ in relation to** **rs8176719 (lead variant)** |
| rs8176749 | 28 | 0.114 |
| rs8176745 | Not reported | 0.199 |
| rs1053878 | 30 | 0.164 |
| rs8176720 | Not reported | 0.026 |
| **rs8176719** | 23,30 | 1.0 |
| rs512770 | Not reported | 0.053 |
| rs8176704 | 30 | 0.186 |
| rs687621 | 24 | 0.868 |
| rs687289 | 28,30 | 0.868 |
| rs2519093 | 23,27,28,30 | 0.369 |
| rs9411377 | 29 | 0.719 |
| rs587611953 | 30 | 0.309 |
| rs582094 | 32 | 0.857 |
| rs8176645 | 27 | 0.977 |
| rs505922 | 30 | 0.869 |
| rs529565 | 25 | 0.857 |
| rs579459 | 28 | 0.433 |
| rs495828 | 23 | 0.433 |
| rs635634 | 30,31 | 0.362 |
| rs9411395 | 30 | 0.069 |
| r^2^ is the square of the correlation between a pair of loci [66]. | | |

| **Supplementary Table 5**. Linkage disequilibrium (LD) expressed as R^2^ for *VWF* gene variants determined using Ldlink [66]. The lead variant **rs1063856 (p.Thr789Ala)** is displayed in bold. | | | | | | | | | | |
| --- | --- | --- | --- | --- | --- | --- | --- | --- | --- | --- |
| RS number | Reference* | rs185699757 | rs216311 | rs57950734 | rs1063857 | **rs1063856** | rs1558519 | rs216296 | rs7135039 | rs1800379 |
| rs185699757 | 30 | 1.0 | 0.009 | NA | 0.002 | 0.002 | 0.002 | 0.005 | 0.002 | 0.006 |
| rs216311 | 28,30 | 0.009 | 1.0 | NA | 0.082 | 0.082 | 0.082 | 0.118 | 0.062 | 0.015 |
| rs57950734 | 30 | NA | NA | NA | NA | NA | NA | NA | NA | NA |
| rs1063857 | NR | 0.002 | 0.082 | NA | 1.0 | 1.0 | 1.0 | 0.079 | 0.924 | 0.407 |
| **rs1063856** | NR | 0.002 | 0.082 | NA | 1.0 | 1.0 | 1.0 | 0.079 | 0.924 | 0.407 |
| rs1558519 | 28,30 | 0.002 | 0.082 | NA | 1.0 | 1.0 | 1.0 | 0.079 | 0.924 | 0.407 |
| rs216296 | 29 | 0.005 | 0.118 | NA | 0.079 | 0.079 | 0.079 | 1.0 | 0.078 | 0.073 |
| rs7135039 | 30,31 | 0.002 | 0.062 | NA | 0.924 | 0.924 | 0.924 | 0.078 | 1.0 | 0.399 |
| rs1800379 | NR | 0.006 | 0.015 | NA | 0.407 | 0.407 | 0.407 | 0.073 | 0.399 | 1.0 |
| *GWAS reference. NR=not reported. r^2^ is the square of the correlation between a pair of loci [66]. | | | | | | | | | | |

| **Supplementary Table 6**. Populations frequencies (MAF) of rs8176719, rs1800291, and rs1063856 variants in gnomAD v4.1.0 [67]. | | | |
| --- | --- | --- | --- |
|  | ***ABO*** | ***F8*** | ***VWF*** |
|  | **rs8176719**  **frameshift** | **rs1800291**  **p.Asp1260Glu** | **rs1063856**  **p.Thr789Ala** |
| **Amish** | 0.6905 | 0.1281 | 0.3857 |
| **European (Finish)** | 0.4654 | 0.1662 | 0.3572 |
| **South Asian** | 0.4551 | 0.05457 | 0.2484 |
| **East Asian** | 0.4231 | 0.07214 | 0.07161 |
| **Ashkenazi Jewish** | 0.4187 | 0.1412 | 0.2943 |
| **Middle Eastern** | 0.3850 | 0.1386 | 0.2809 |
| **European (non-Finnish)** | 0.3505 | 0.1747 | 0.3702 |
| **Remaining** | 0.3483 | 0.1879 | 0.3514 |
| **African/African American** | 0.3030 | 0.6542 | 0.5793 |
| **Admixed American** | 0.2382 | 0.1819 | 0.2098 |
| **All XX (females)** | 0.3530 | 0.1933 | 0.3595 |
| **All XY (males)** | 0.3632 | 0.1783 | 0.3520 |
| **Total** | 0.3581 | 0.1884 | 0.3558 |
| MAF=minor allele frequency. | | | |

**Supplementary Figure 1**


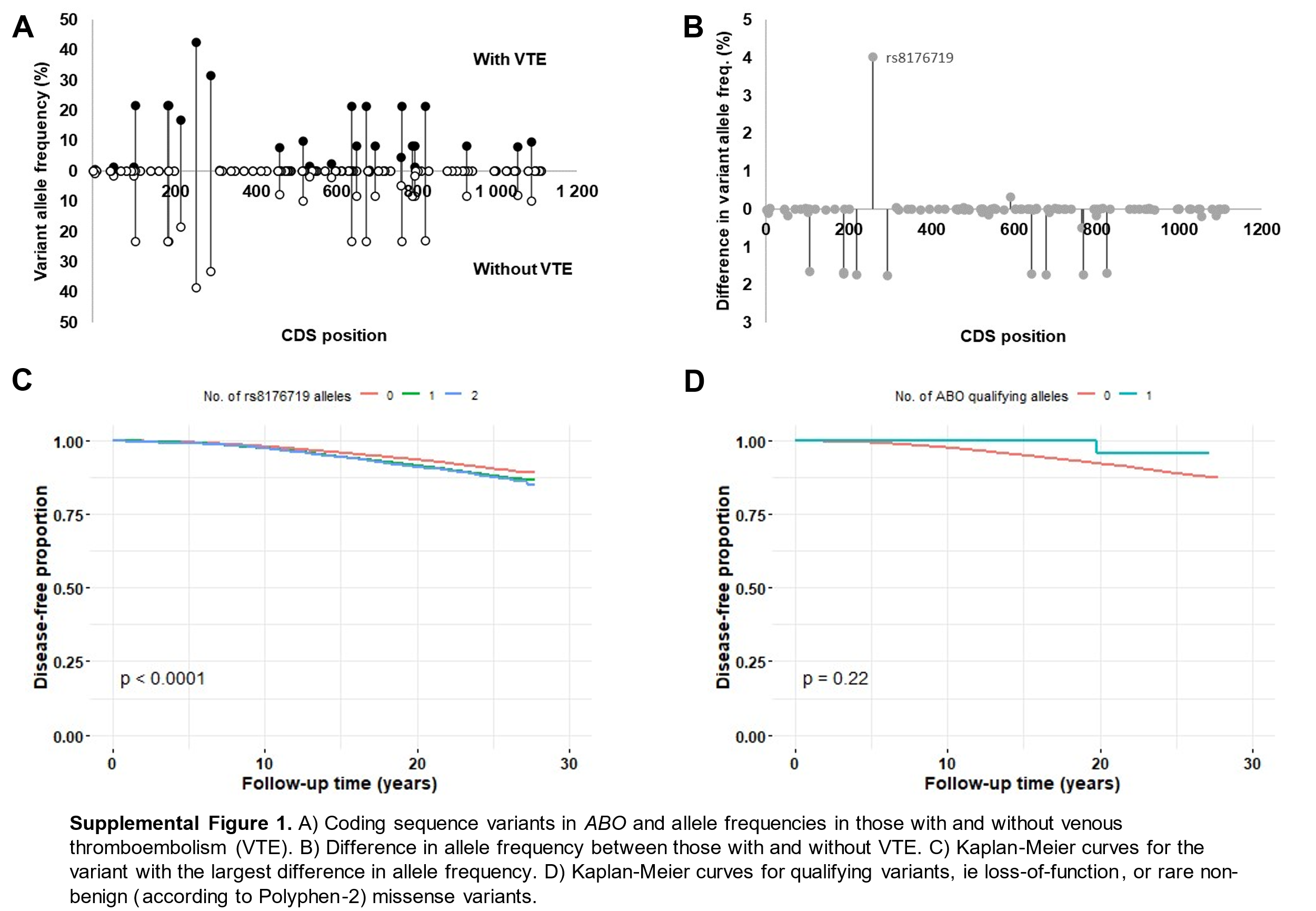


**Supplementary Figure 2**


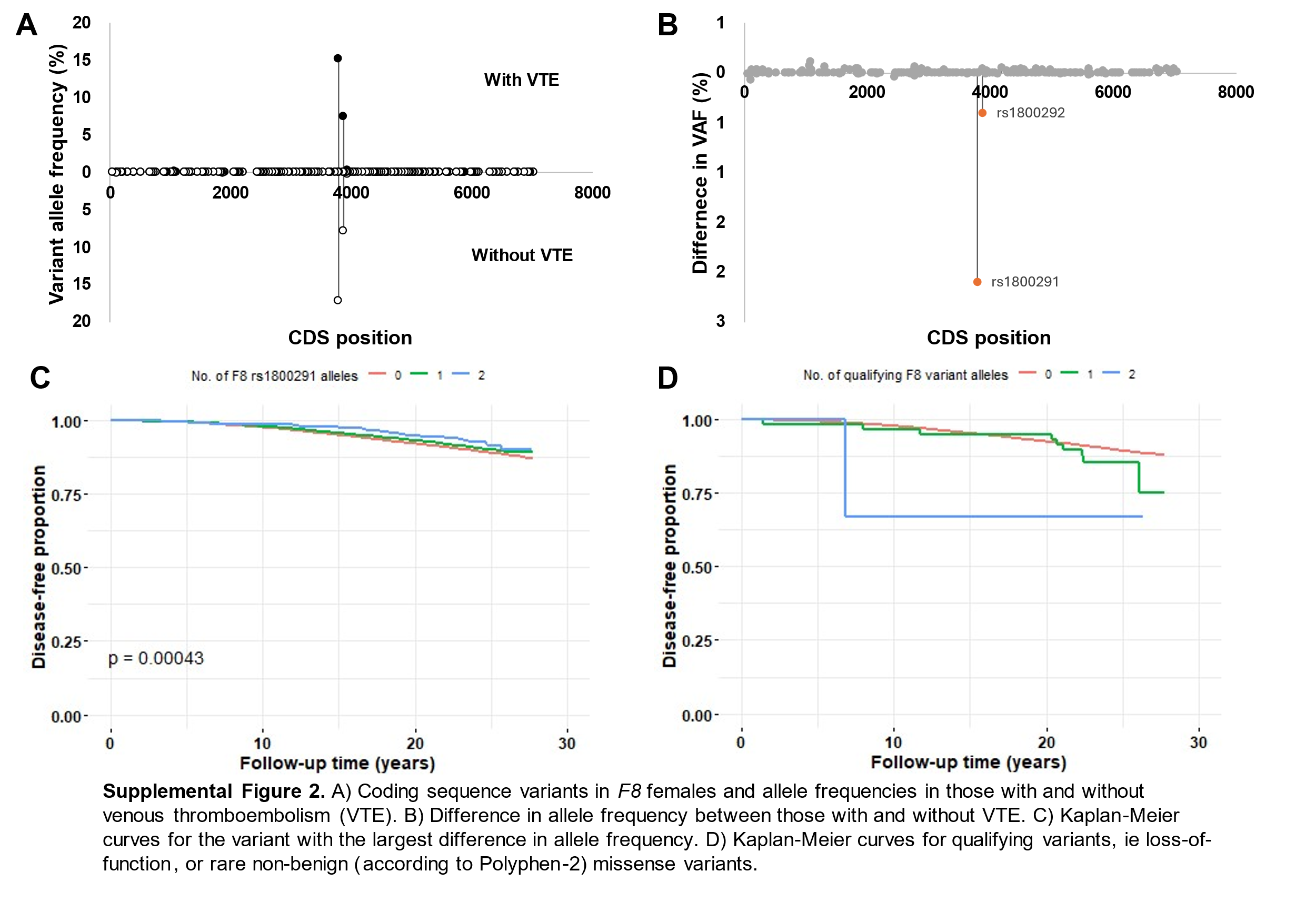


Not significant

**Supplementary Figure 3**


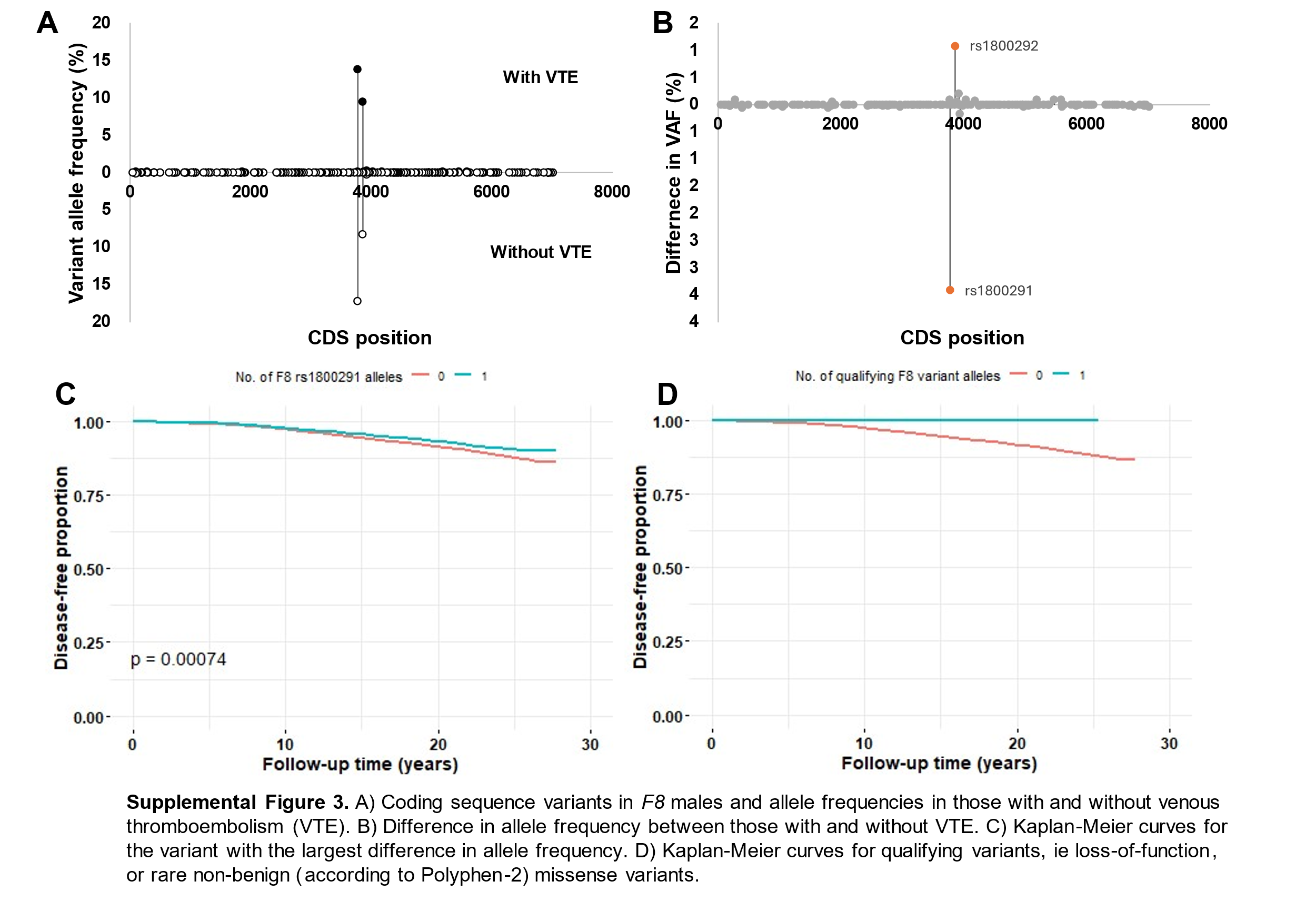


Not significant

**Supplementary figure 4**


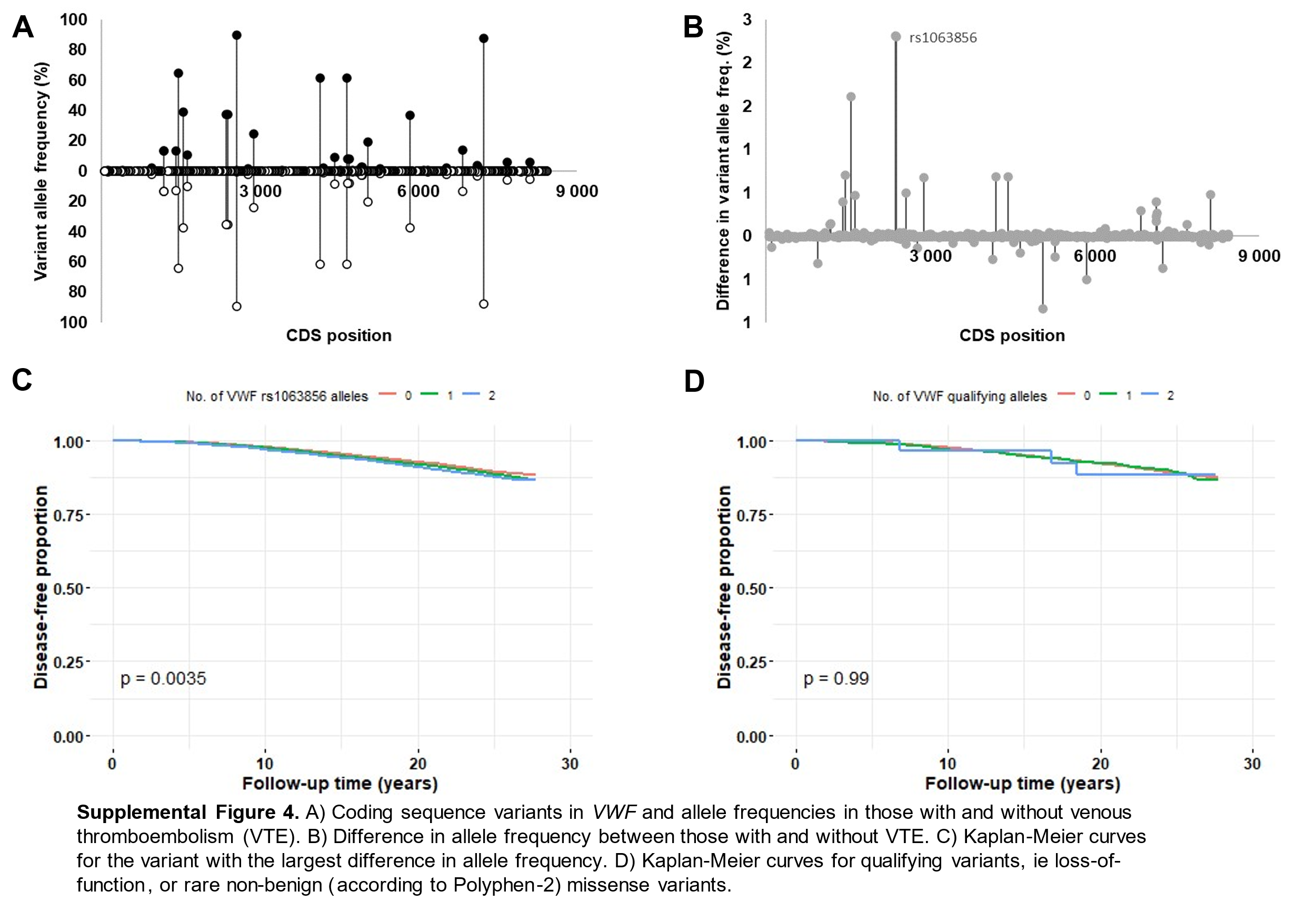


**Supplementary figure 5**


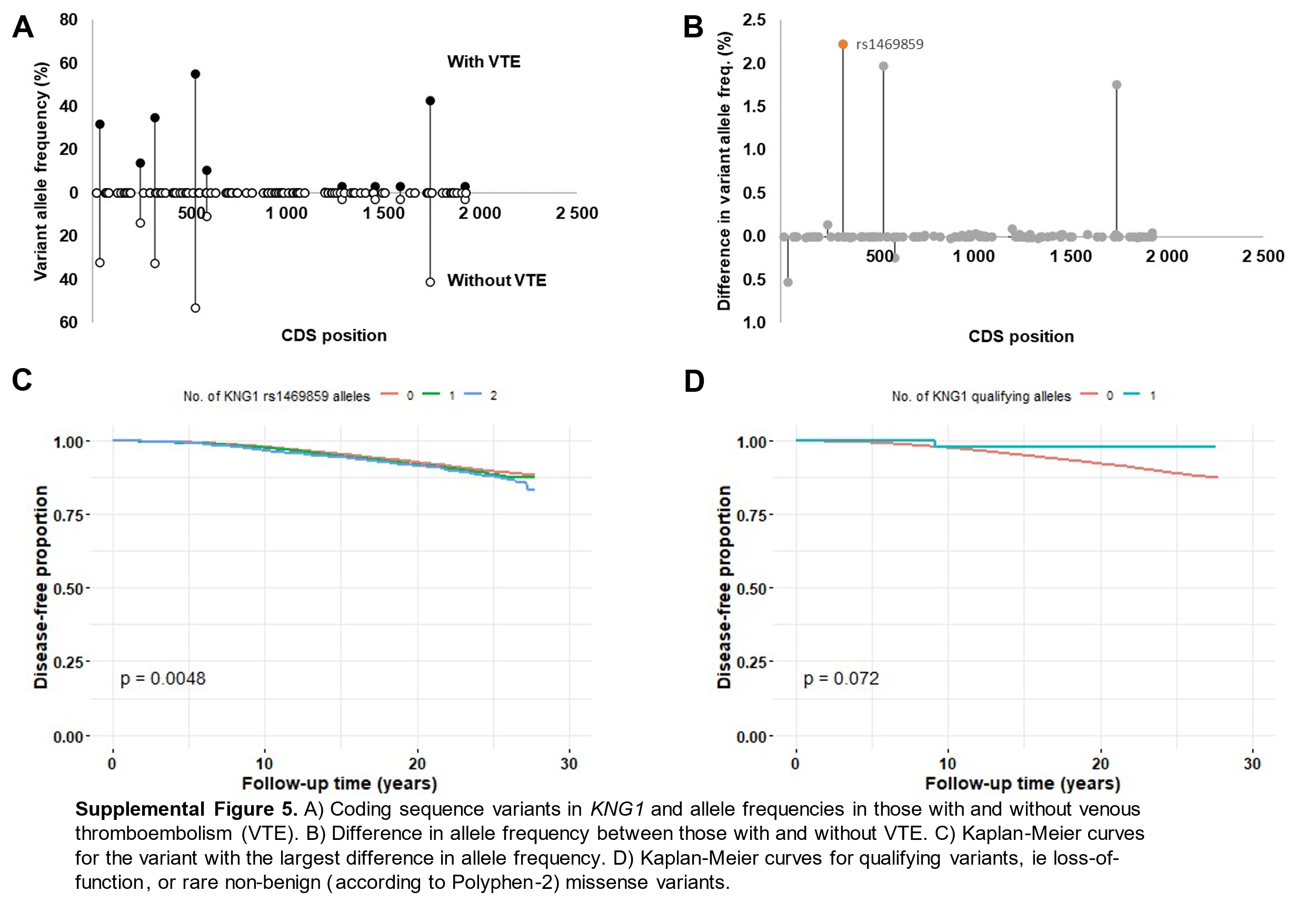


**Supplementary figure 6**


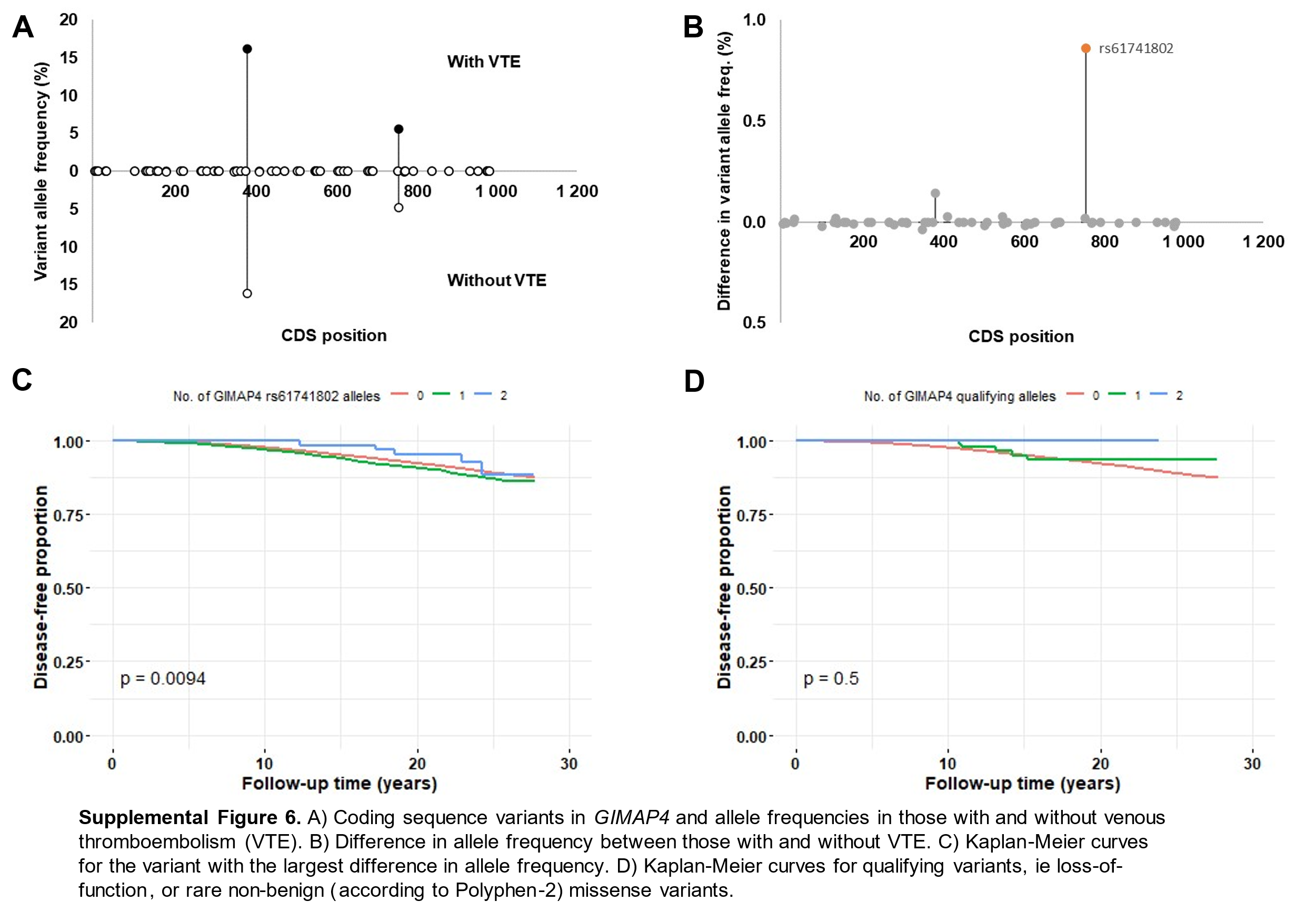


**Supplementary figure 7**


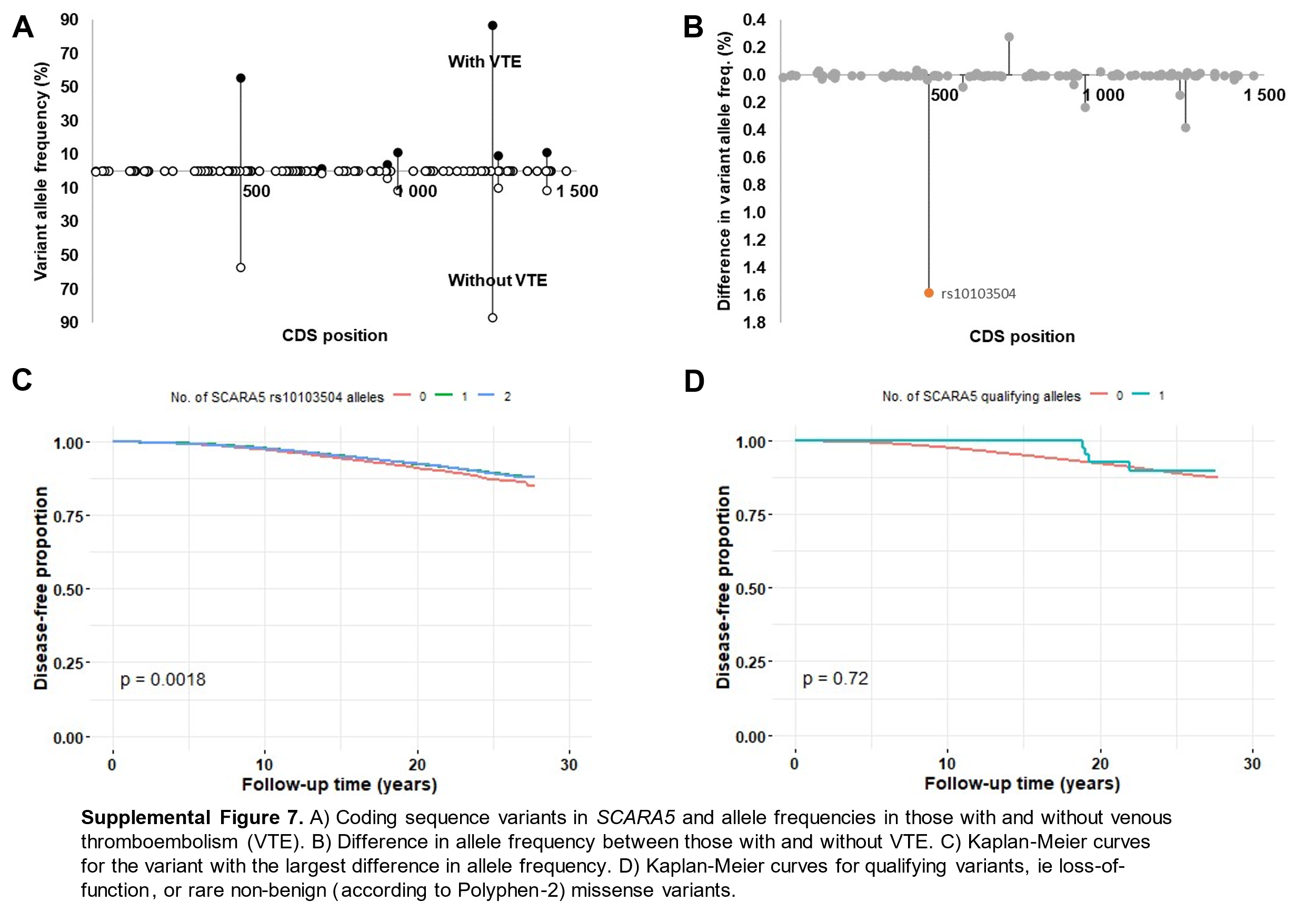


**Supplementary figure 8**


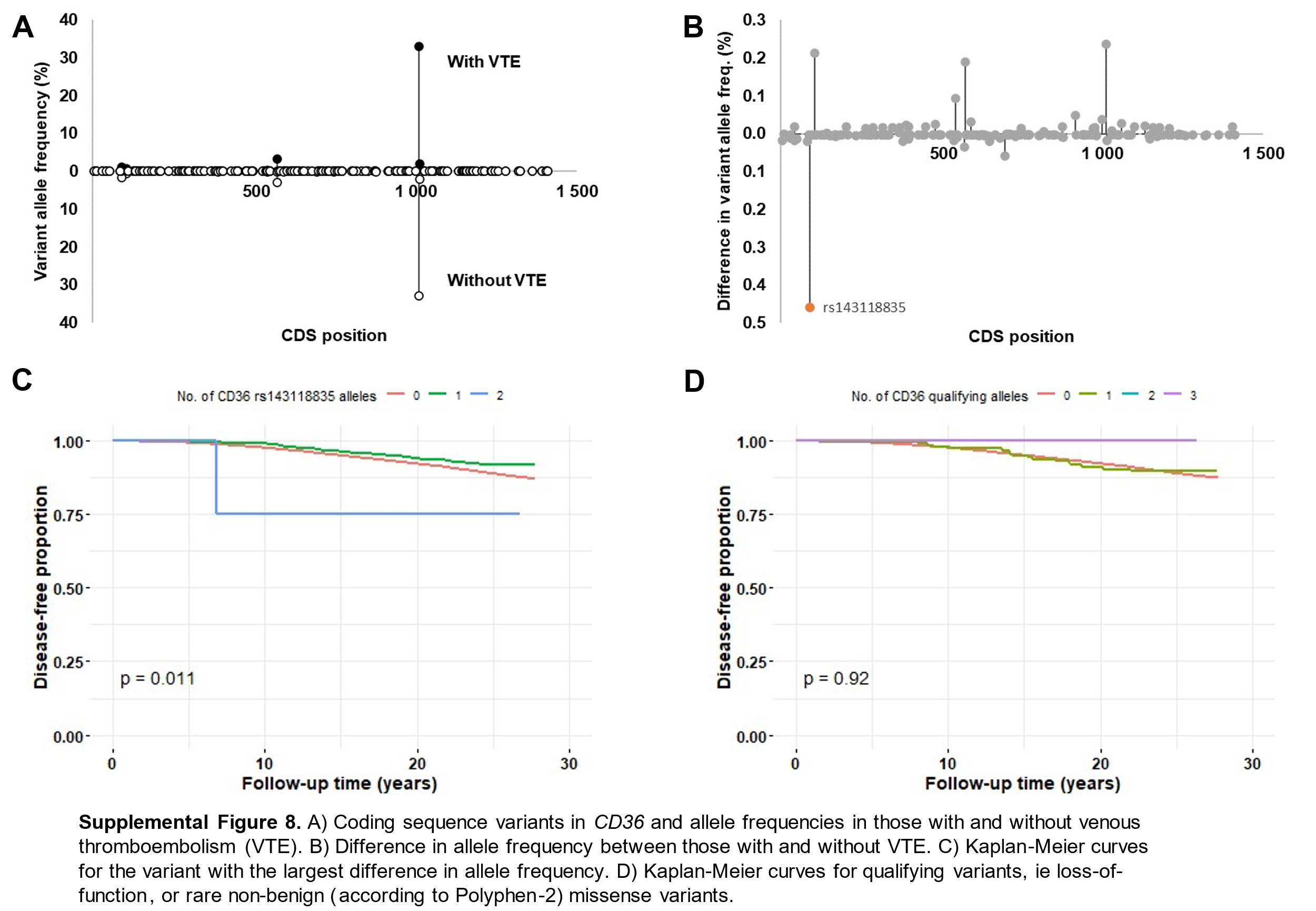


**Supplementary figure 9**


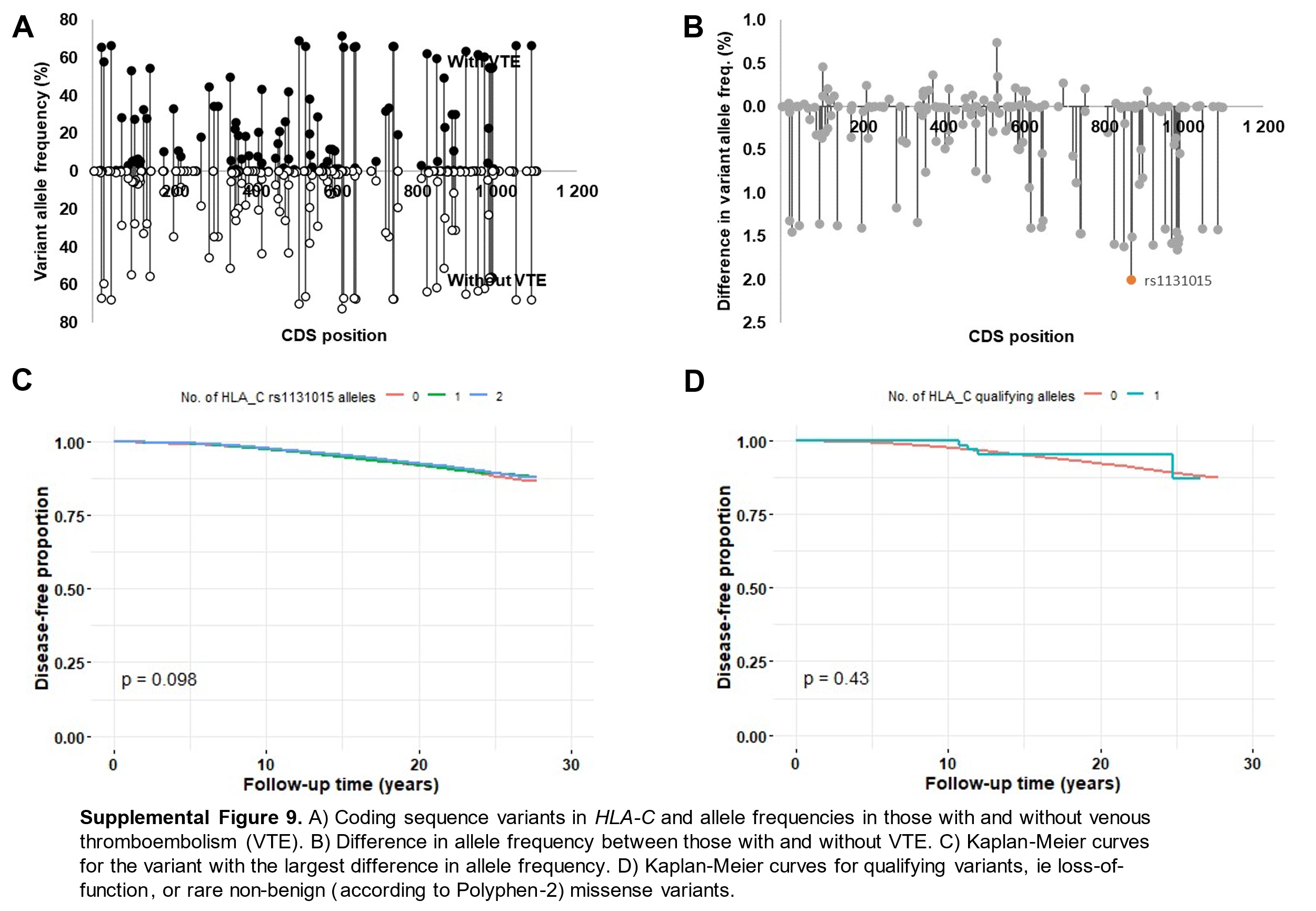


**Supplementary figure 10**


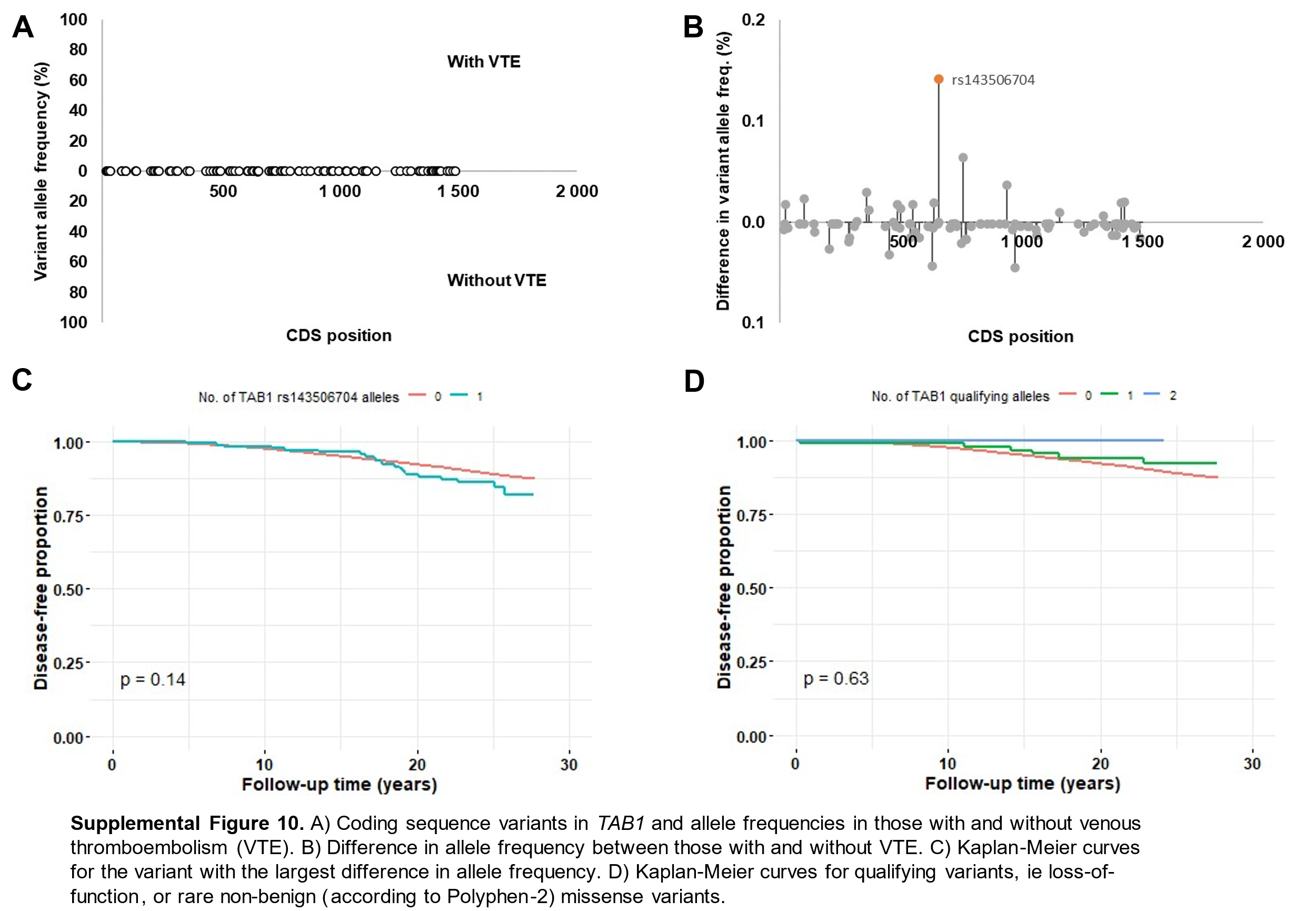


**Supplementary figure 11**


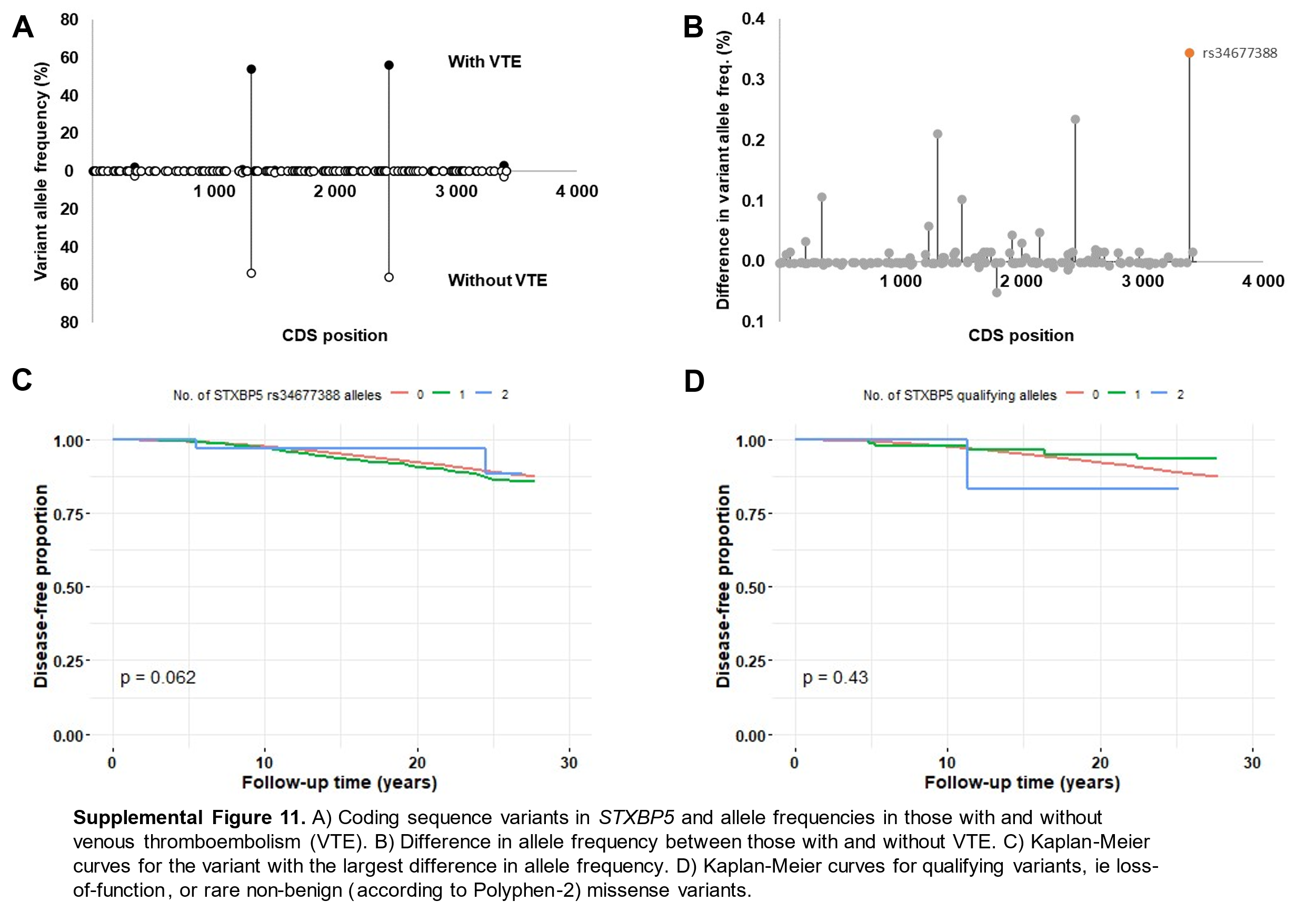


**Supplementary figure 12**


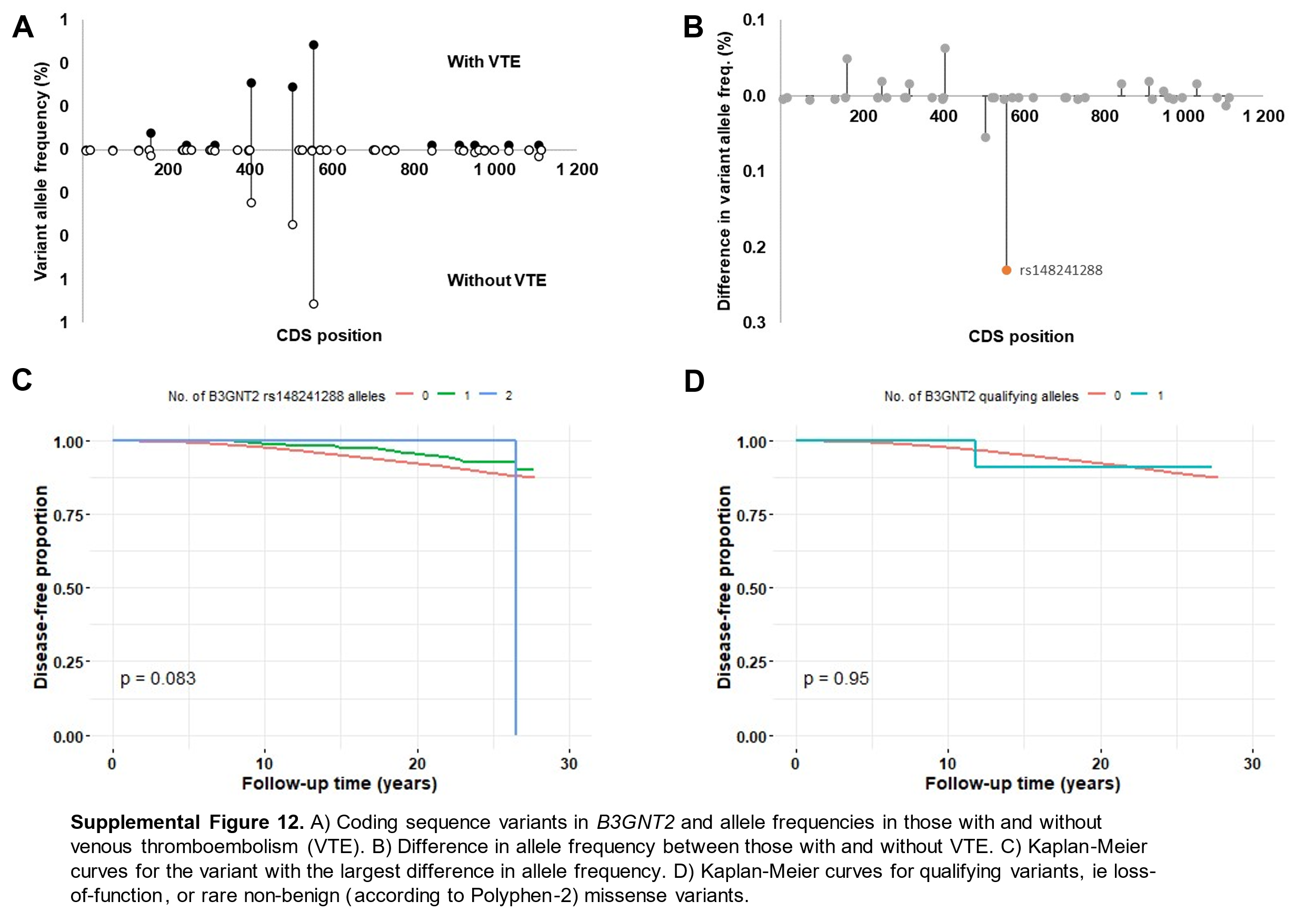


**Supplementary figure 13**


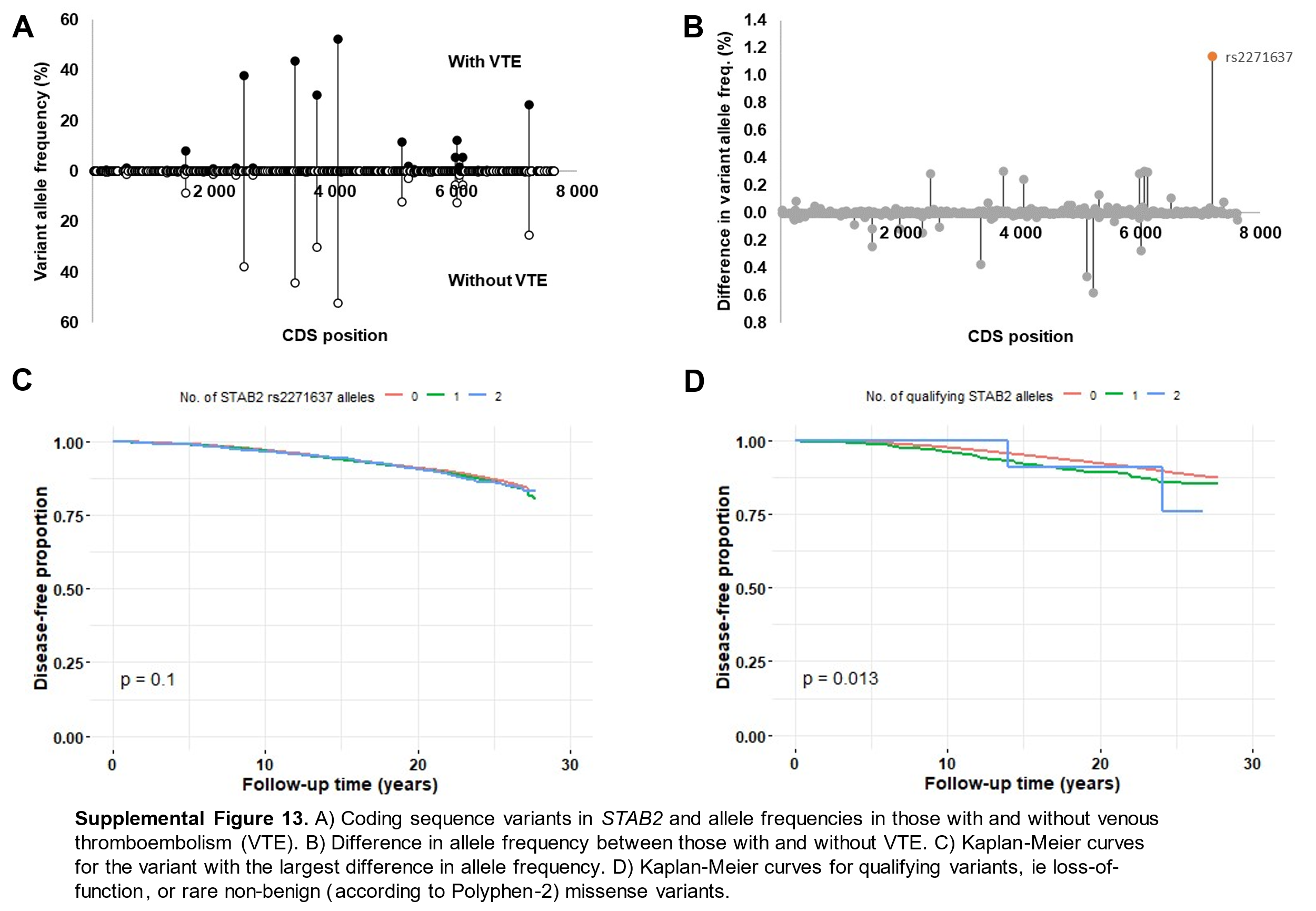


**Supplementary figure 14**


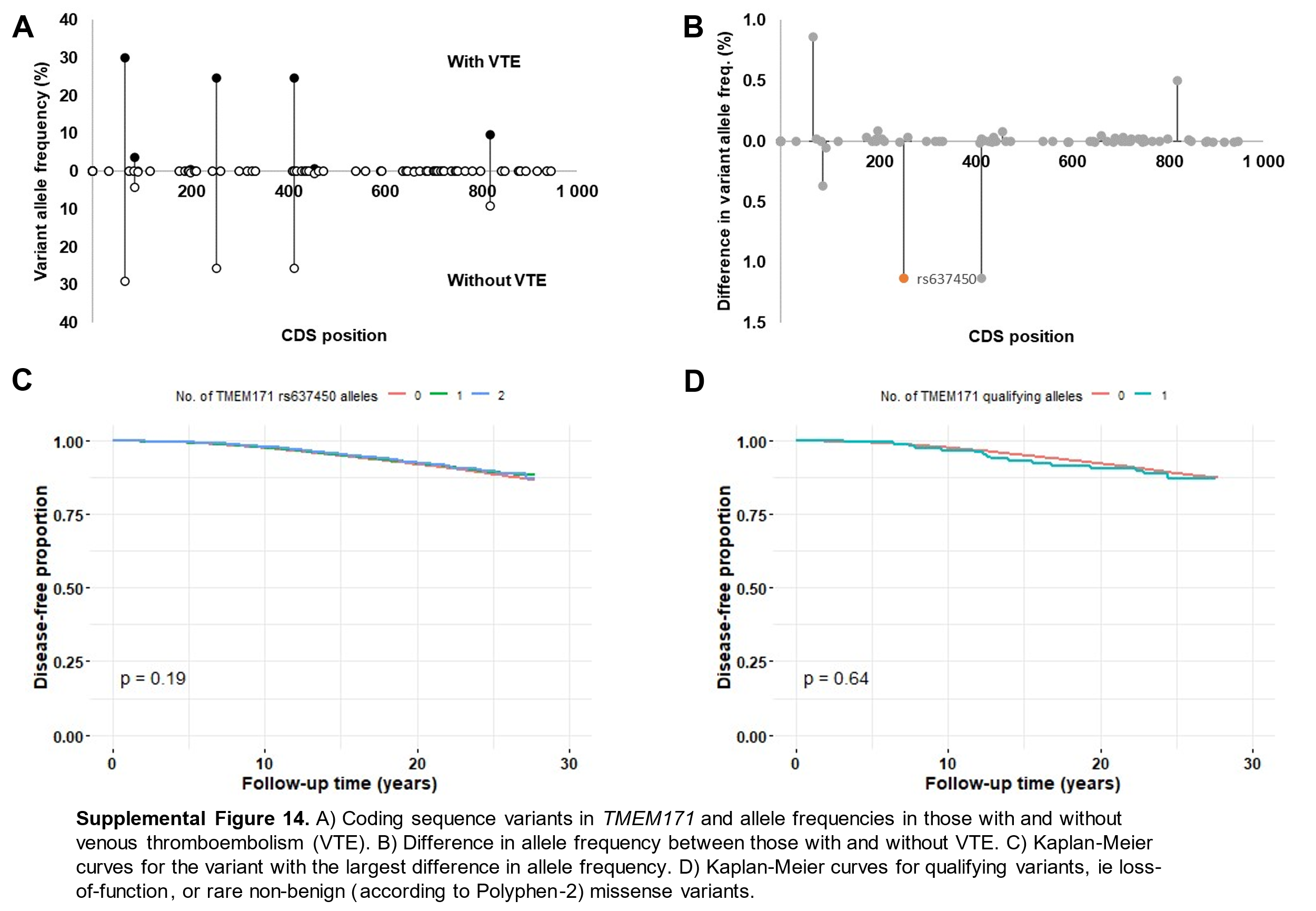


**Supplementary figure 15**


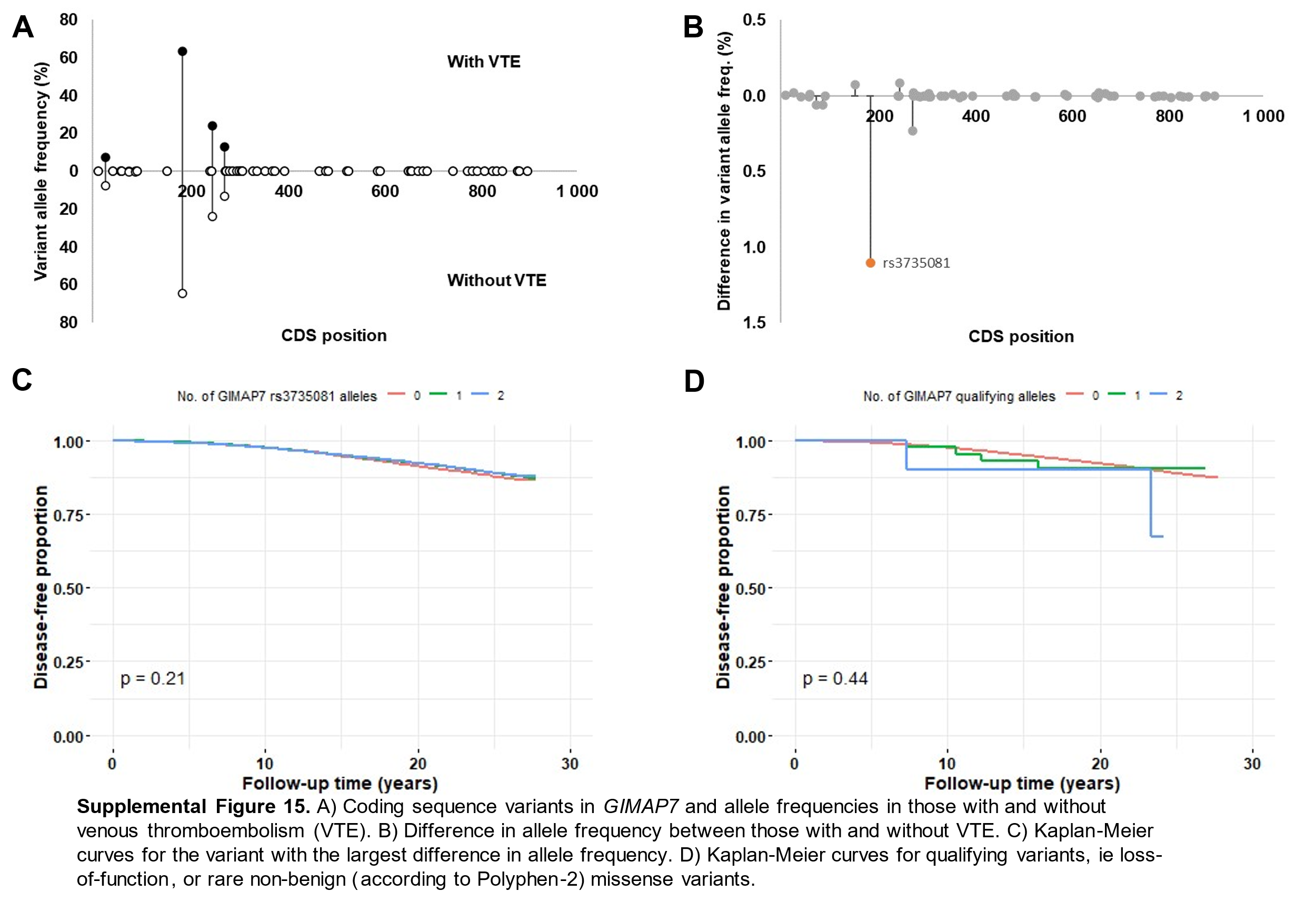


**Supplementary figure 16**


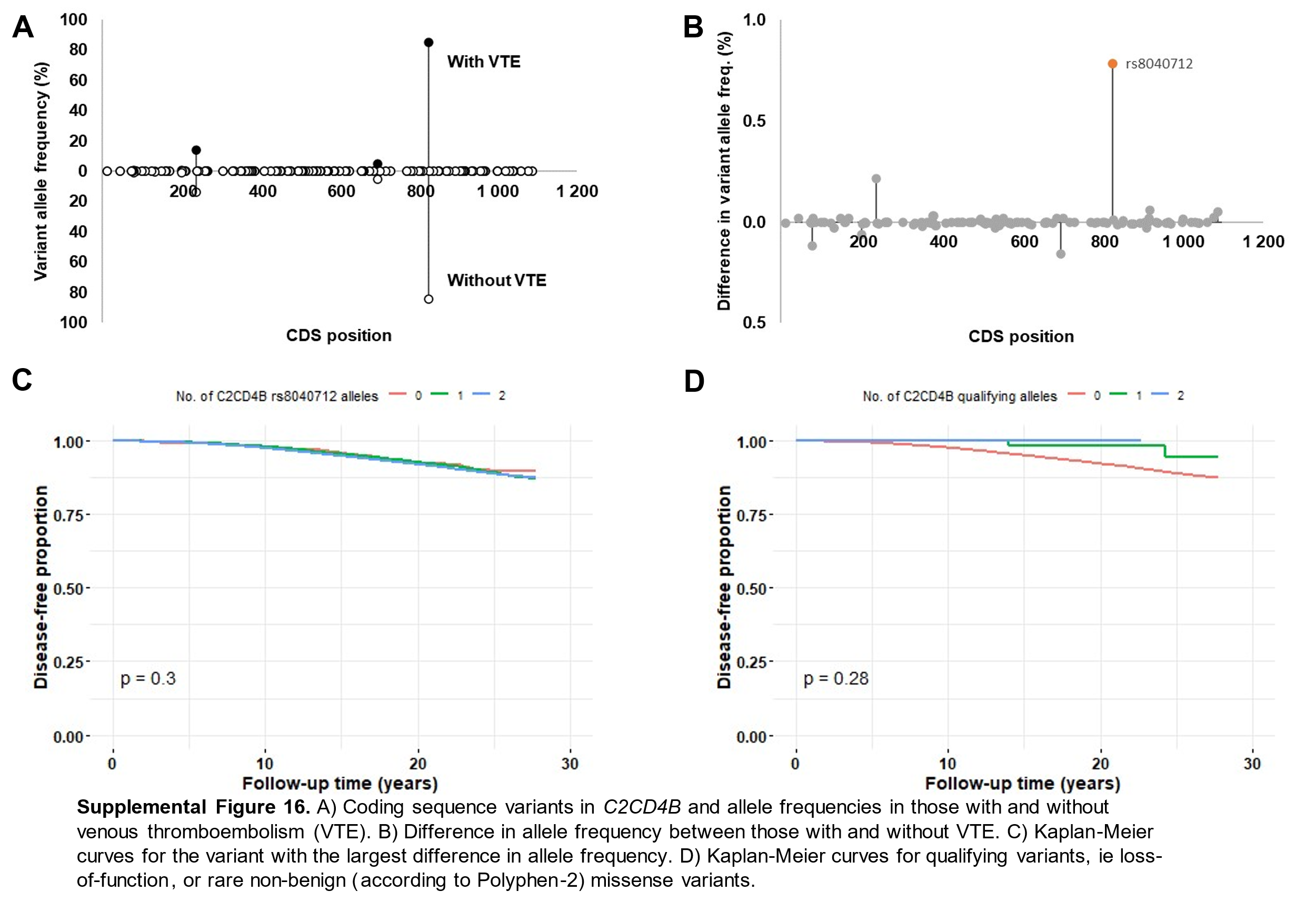


**Supplementary figure 17**


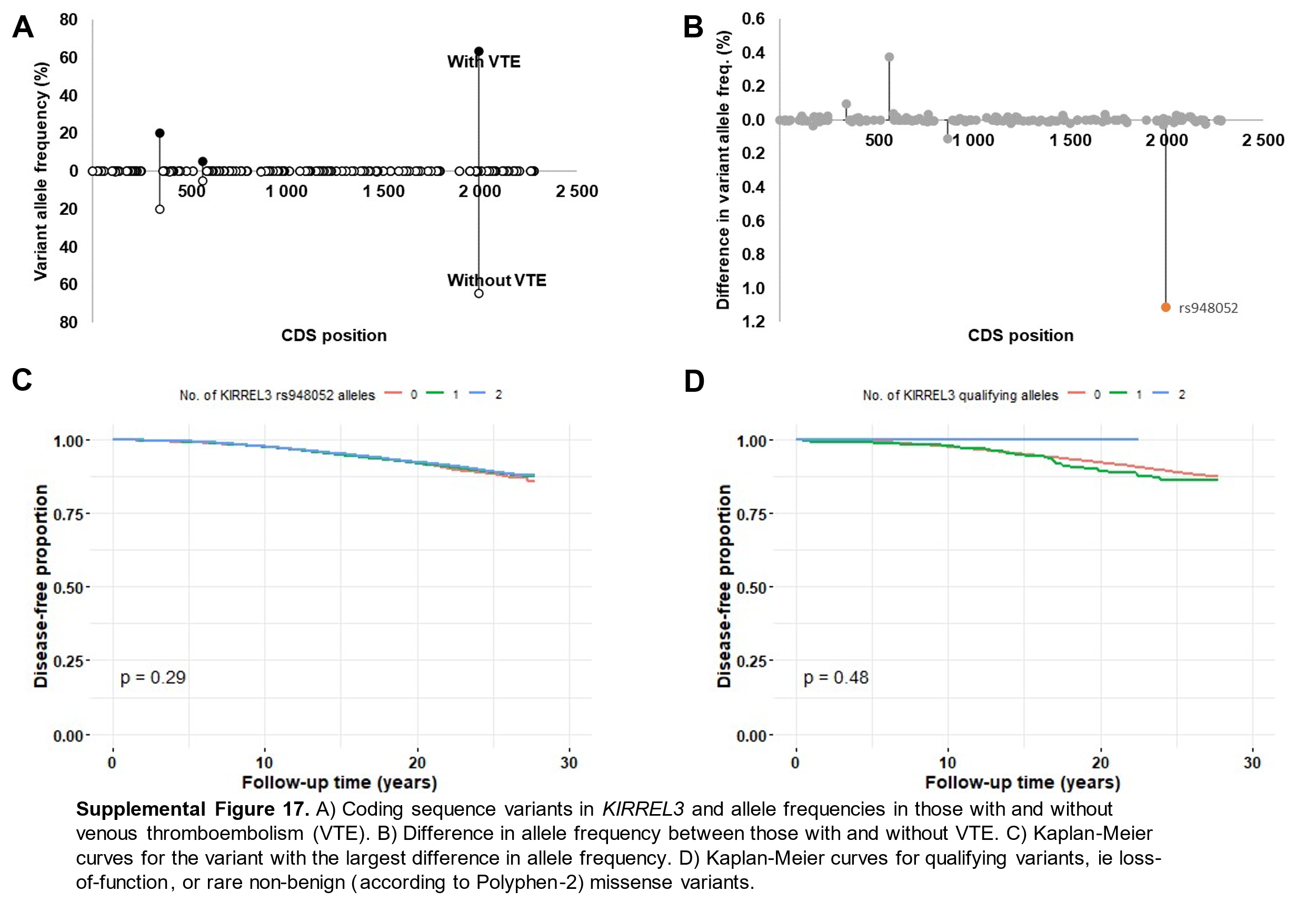


**Supplementary figure 18**


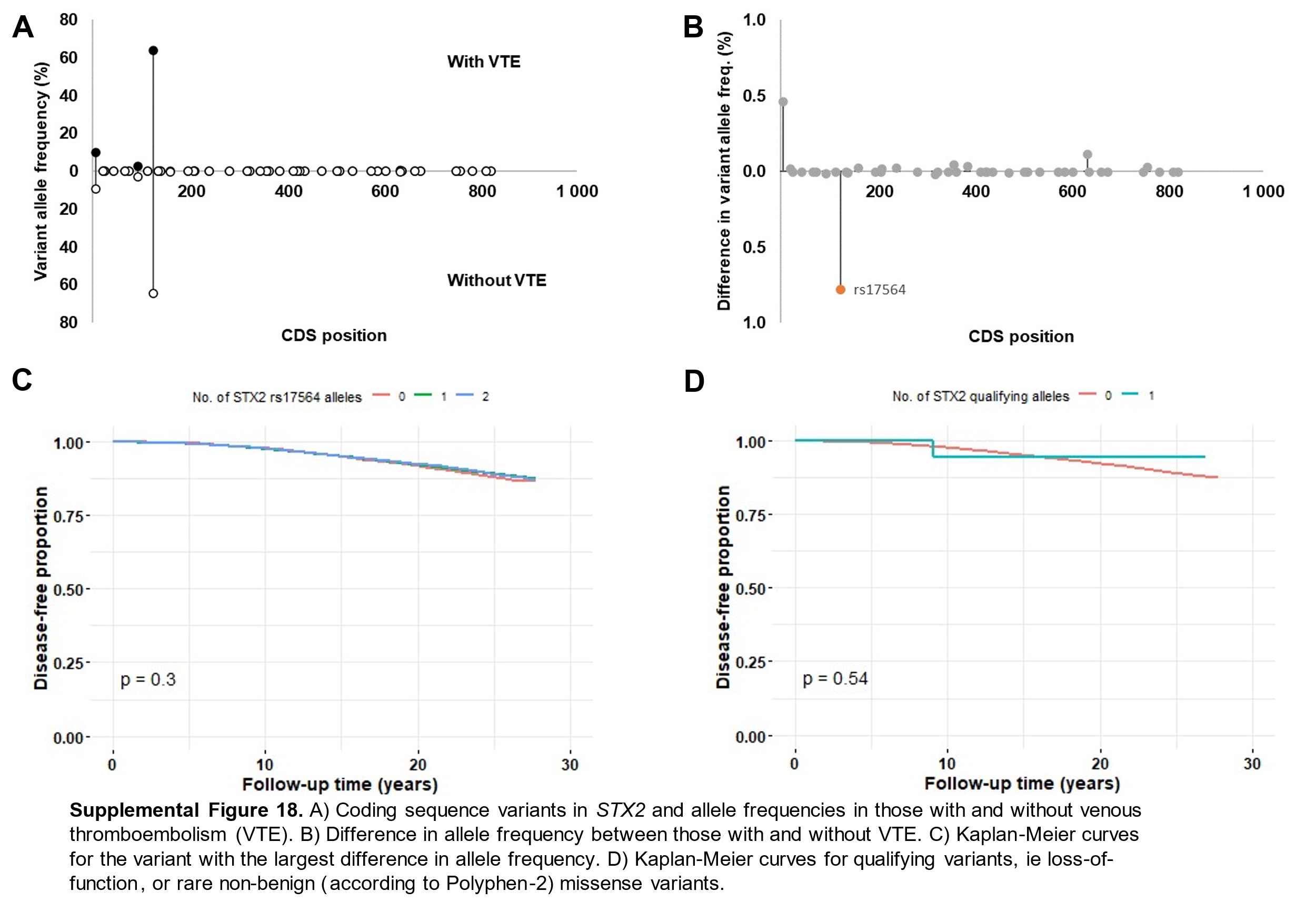


**Supplementary figure 19**


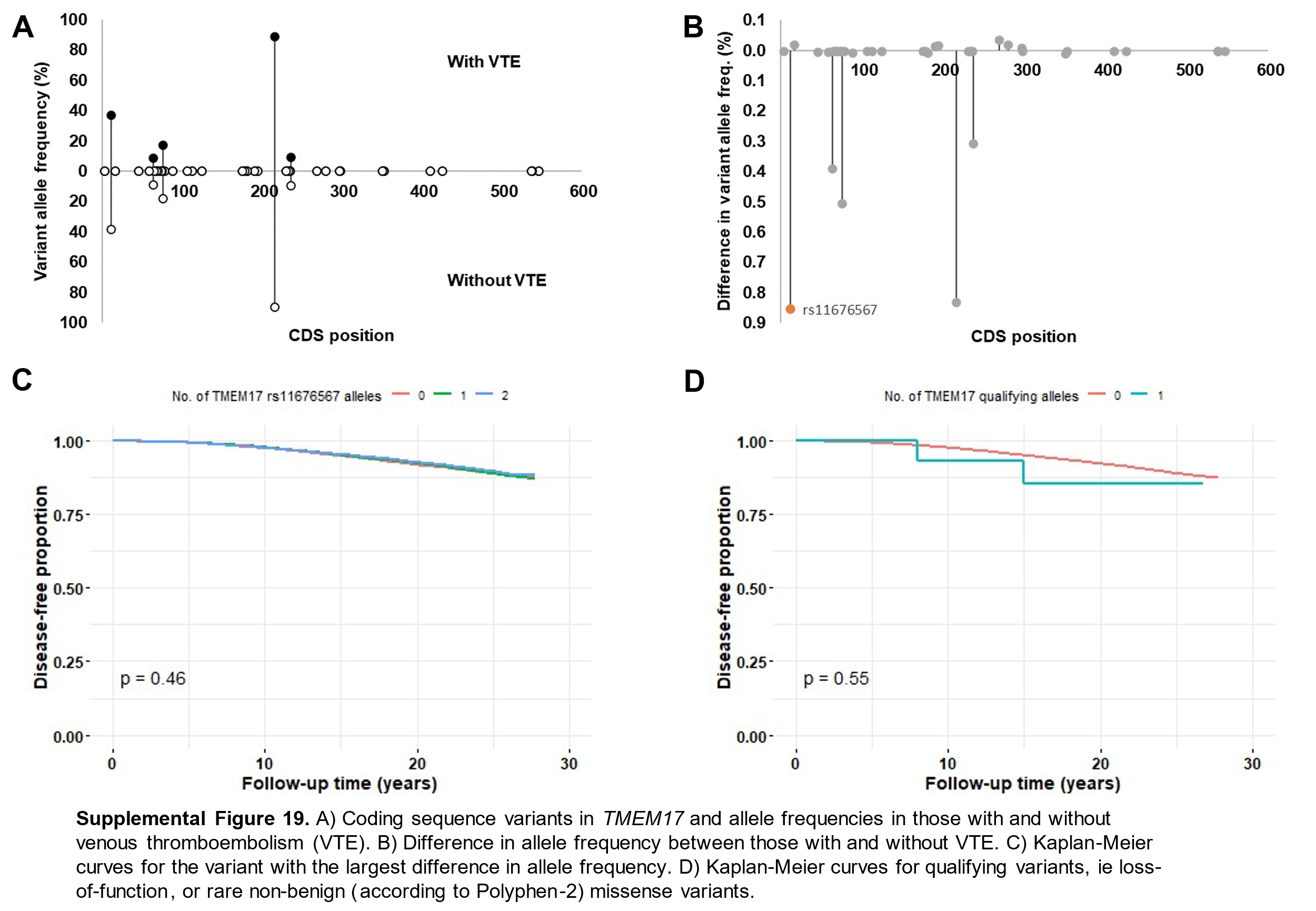


**Supplementary figure 20**


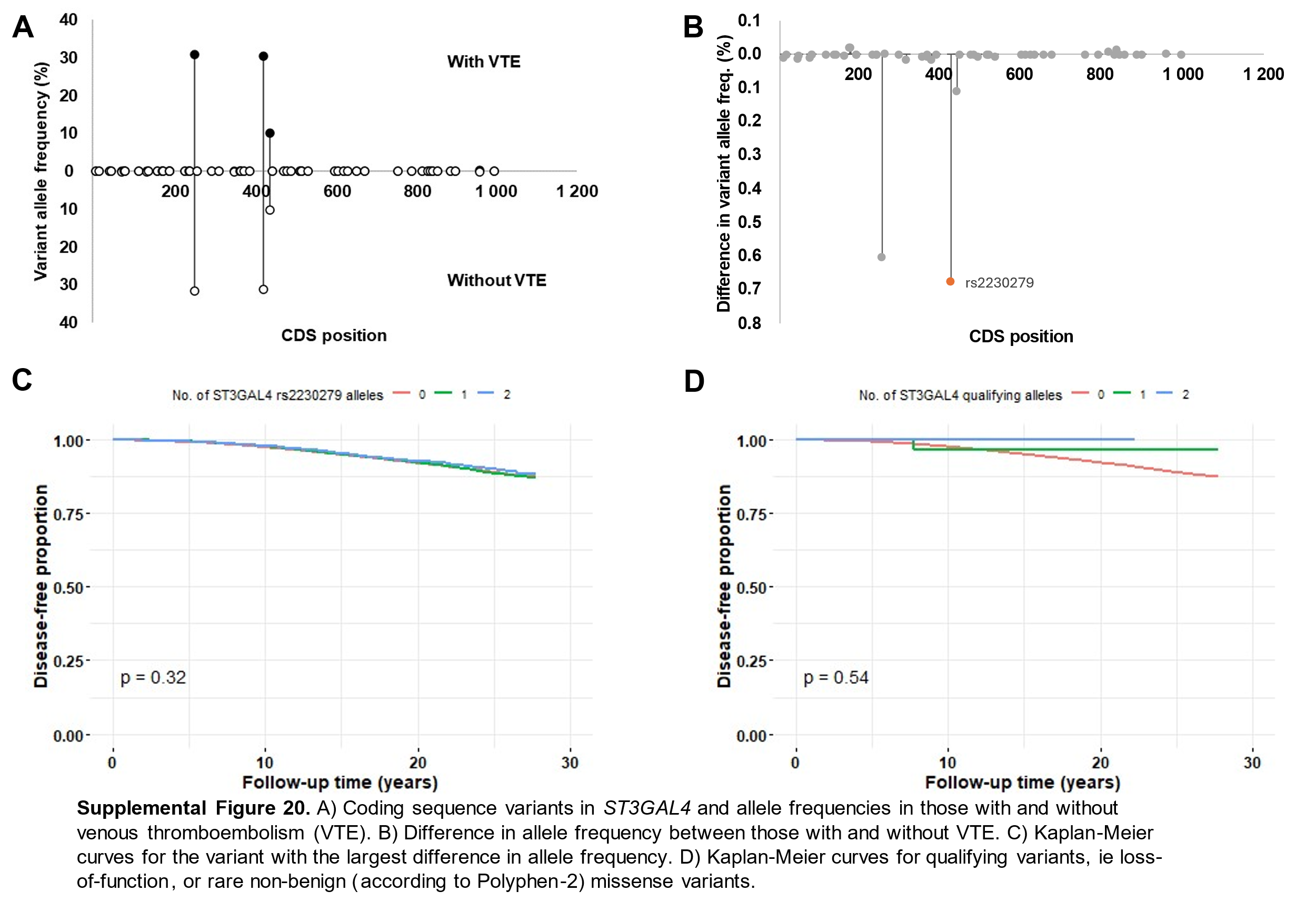


**Supplementary figure 21**


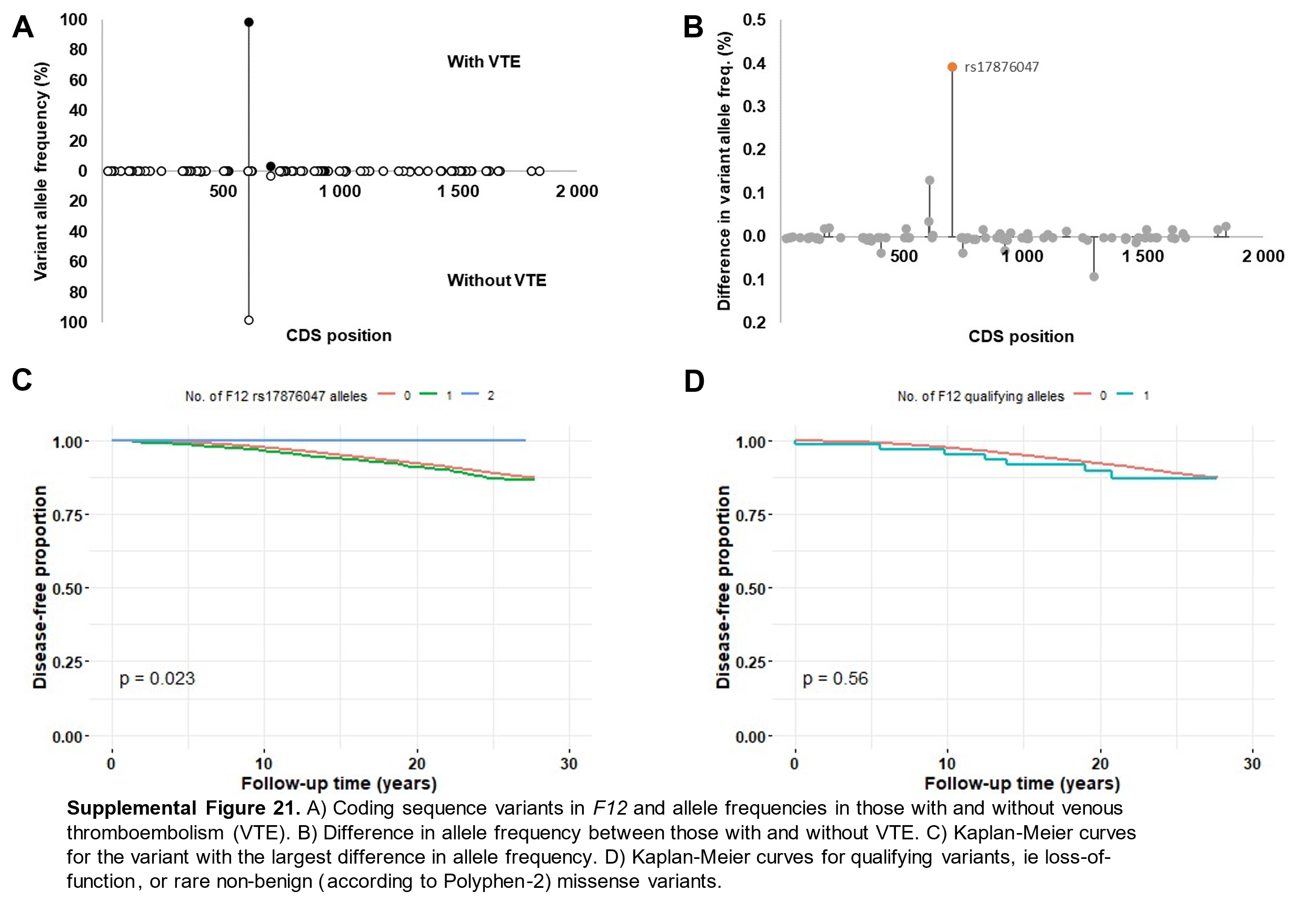


**Supplementary figure 22**


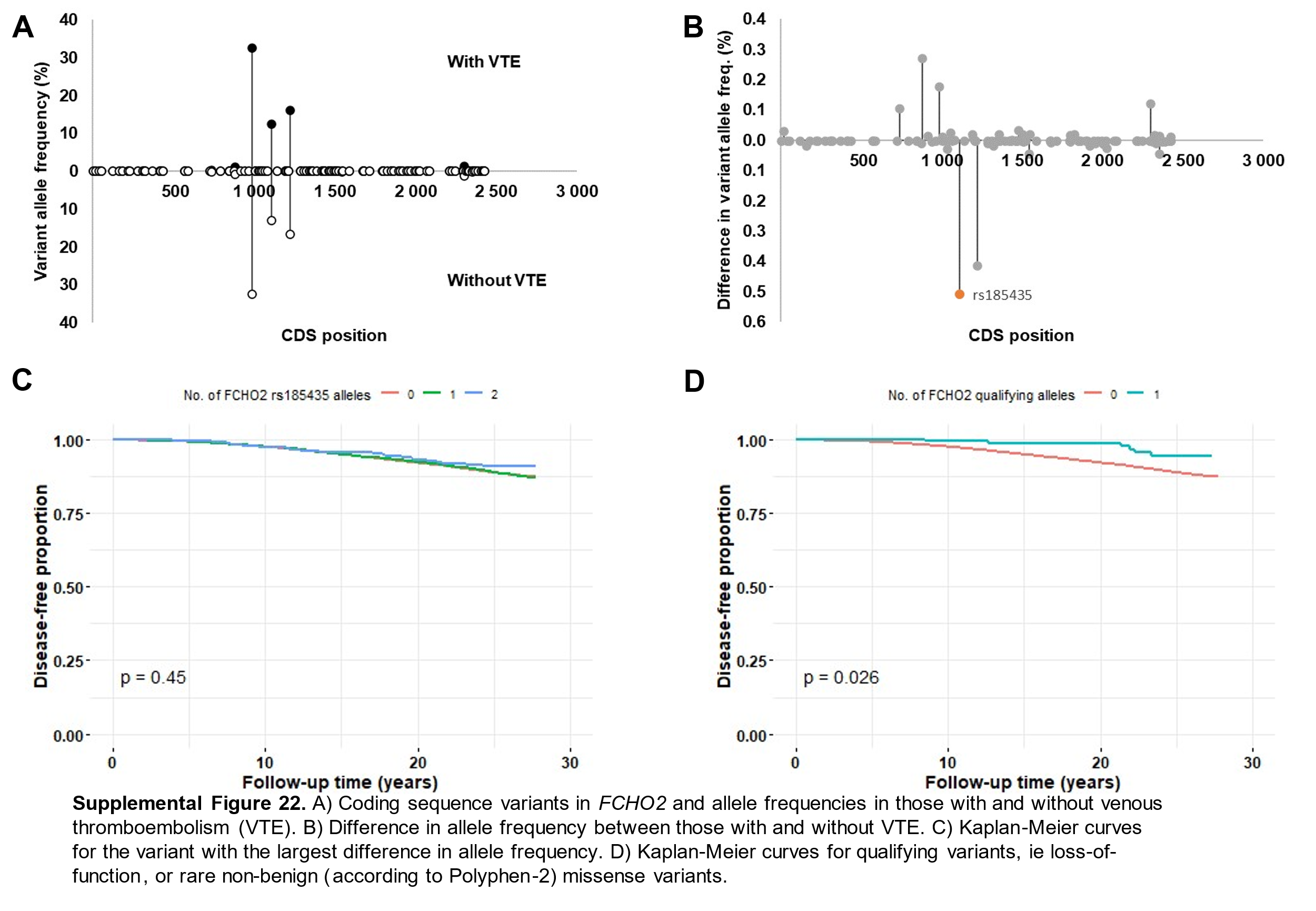


**Supplementary figure 23**


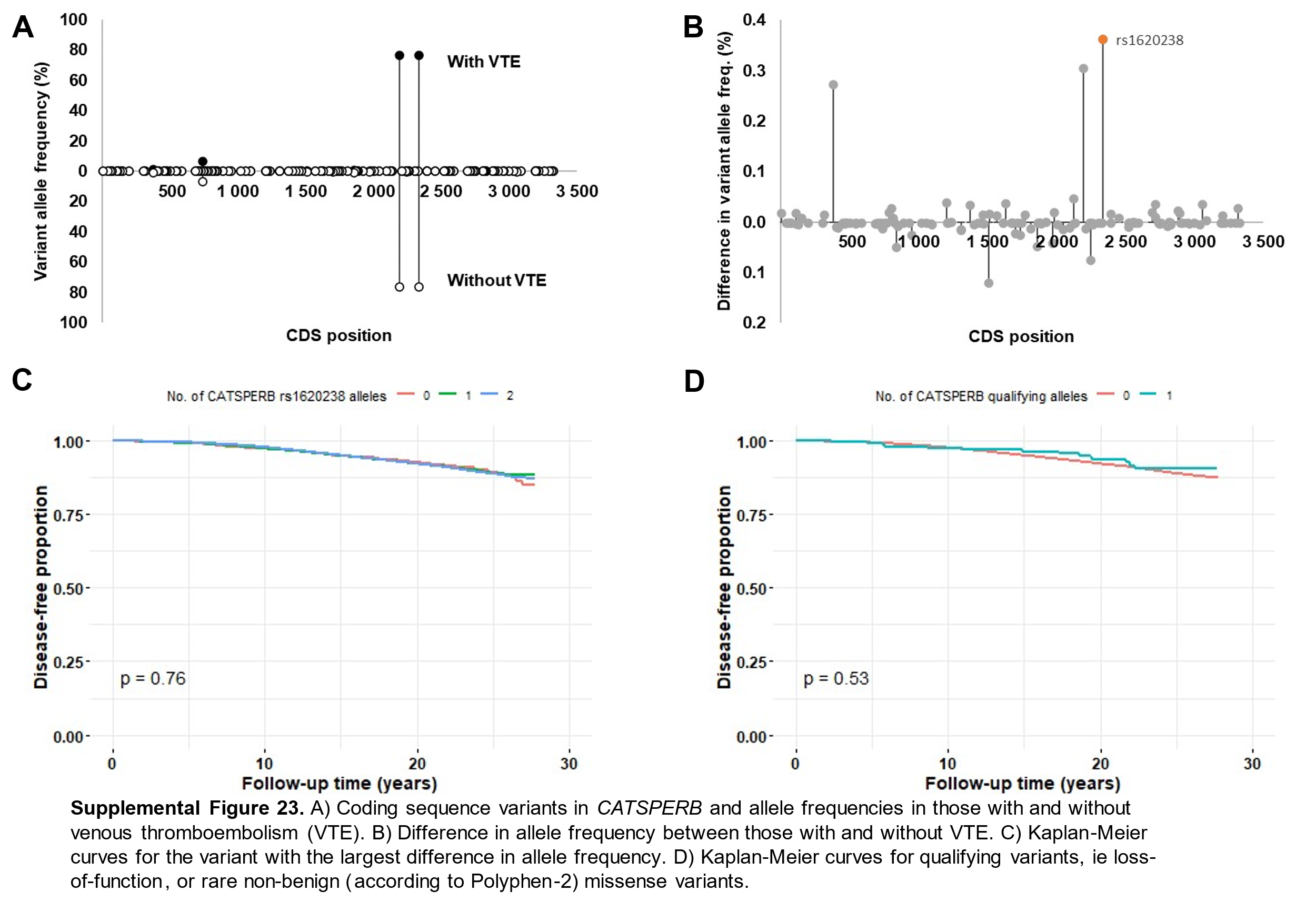


**Supplementary figure 24**


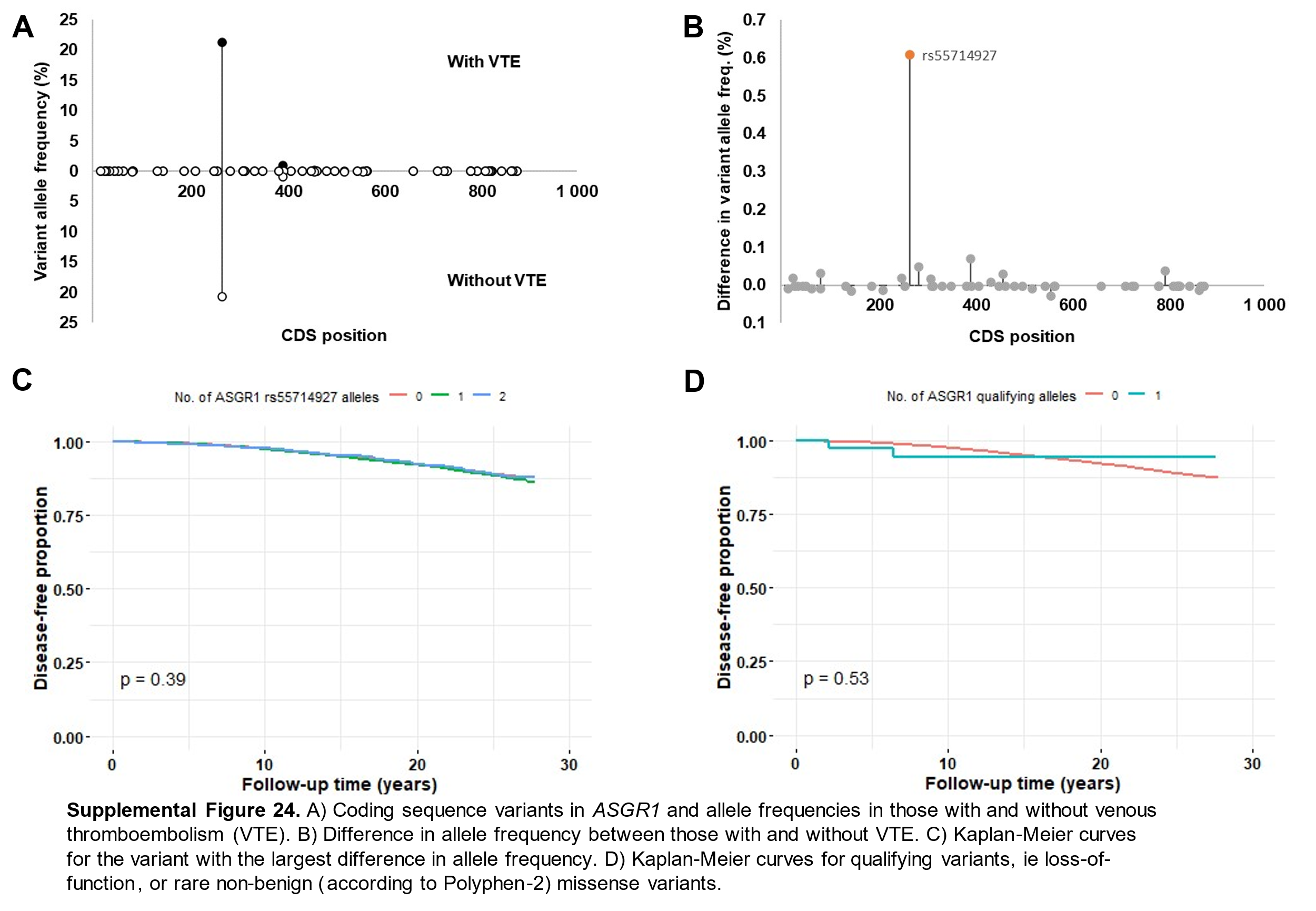


**Supplementary figure 25**


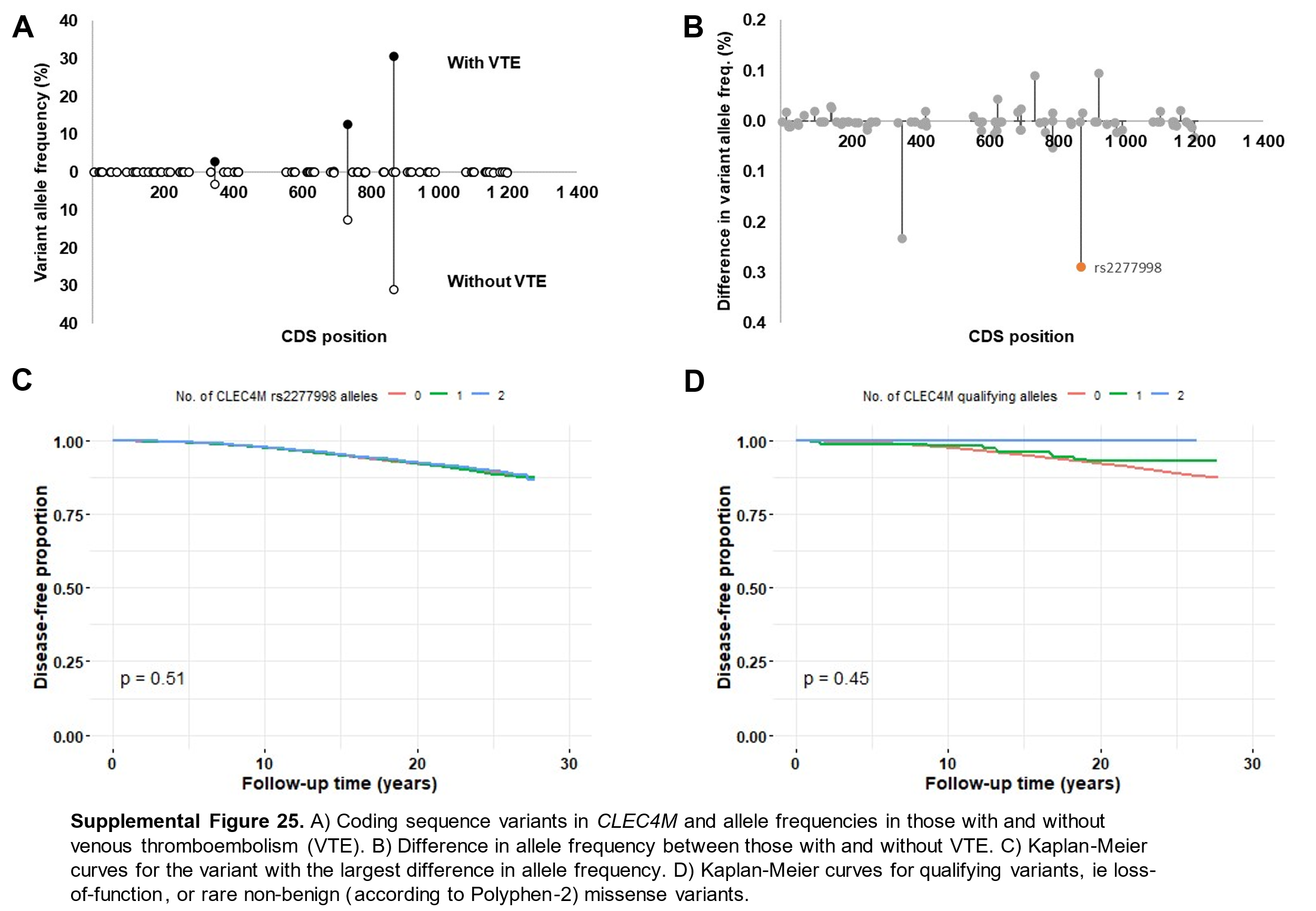


**Supplementary figure 26**


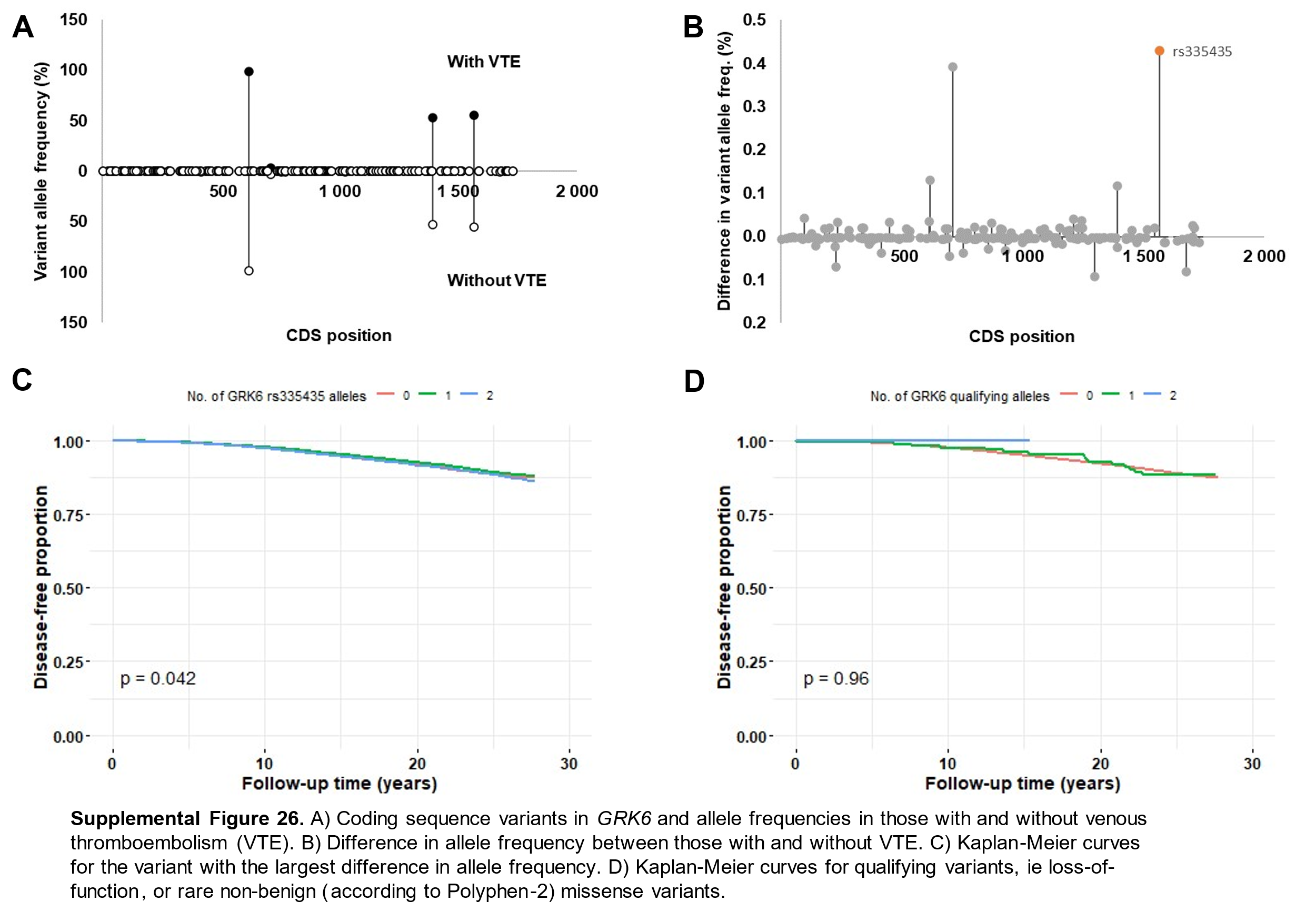


**Supplementary figure 27**


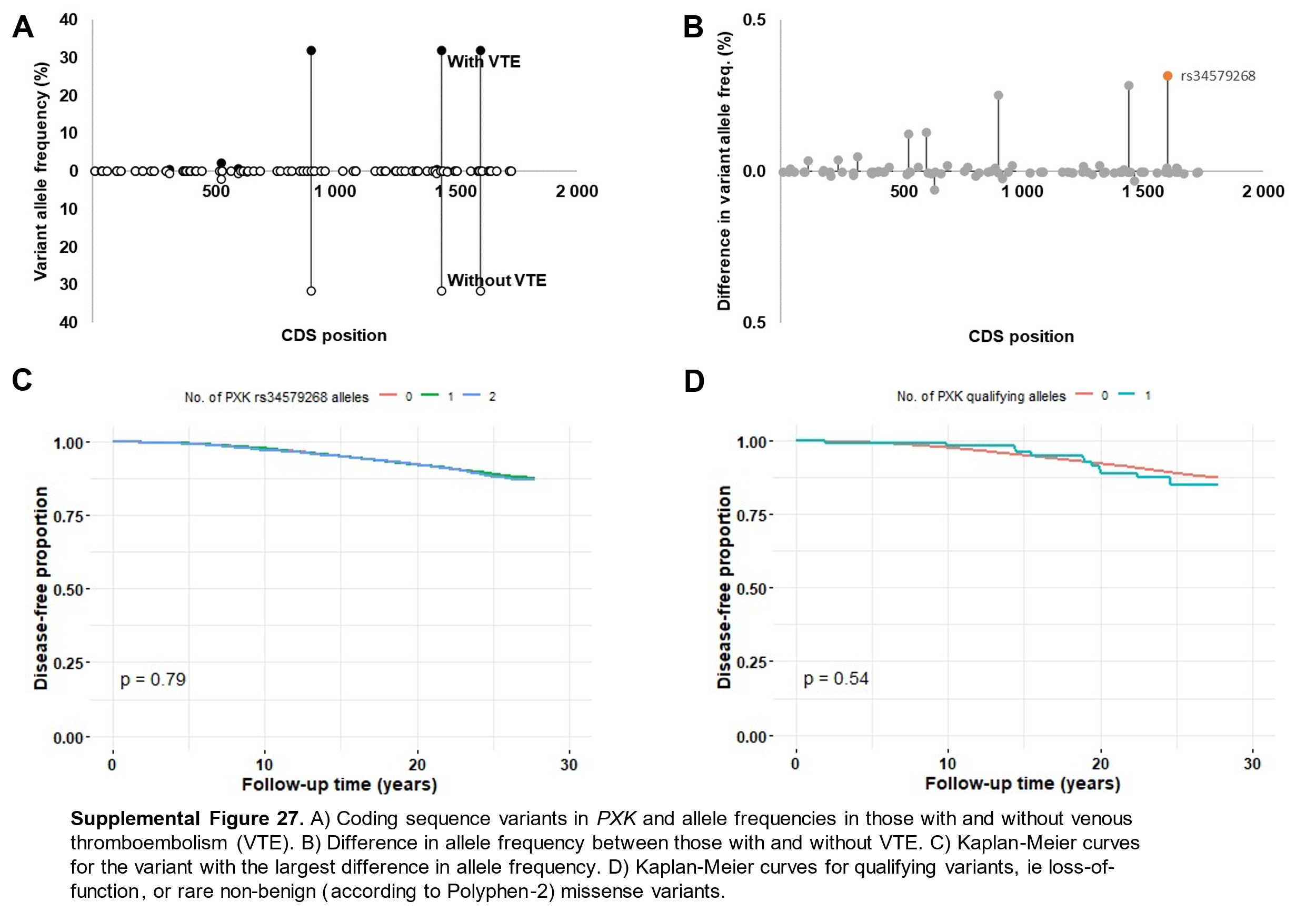


**Supplementary figure 28**


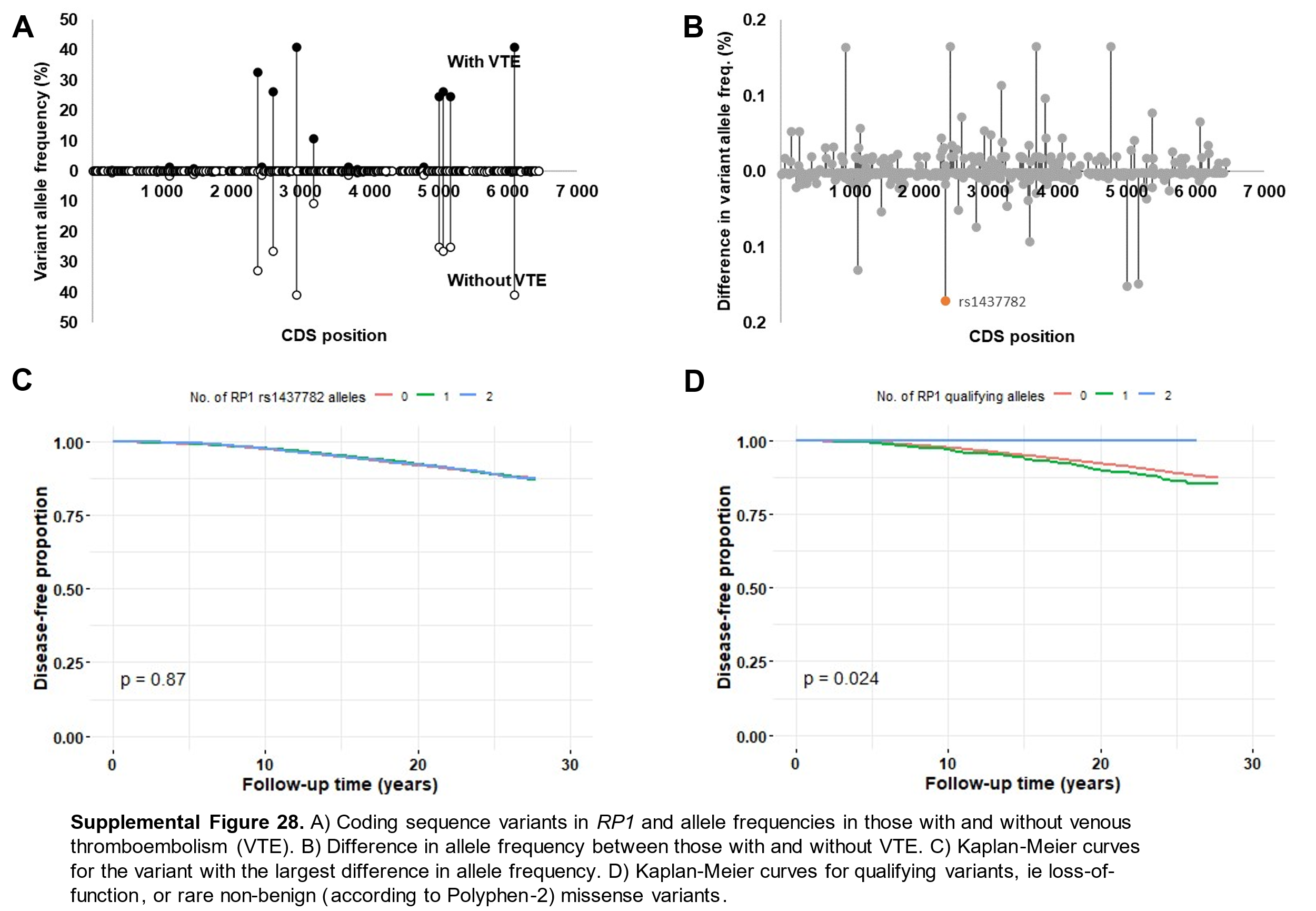


**Supplementary Figure 29**


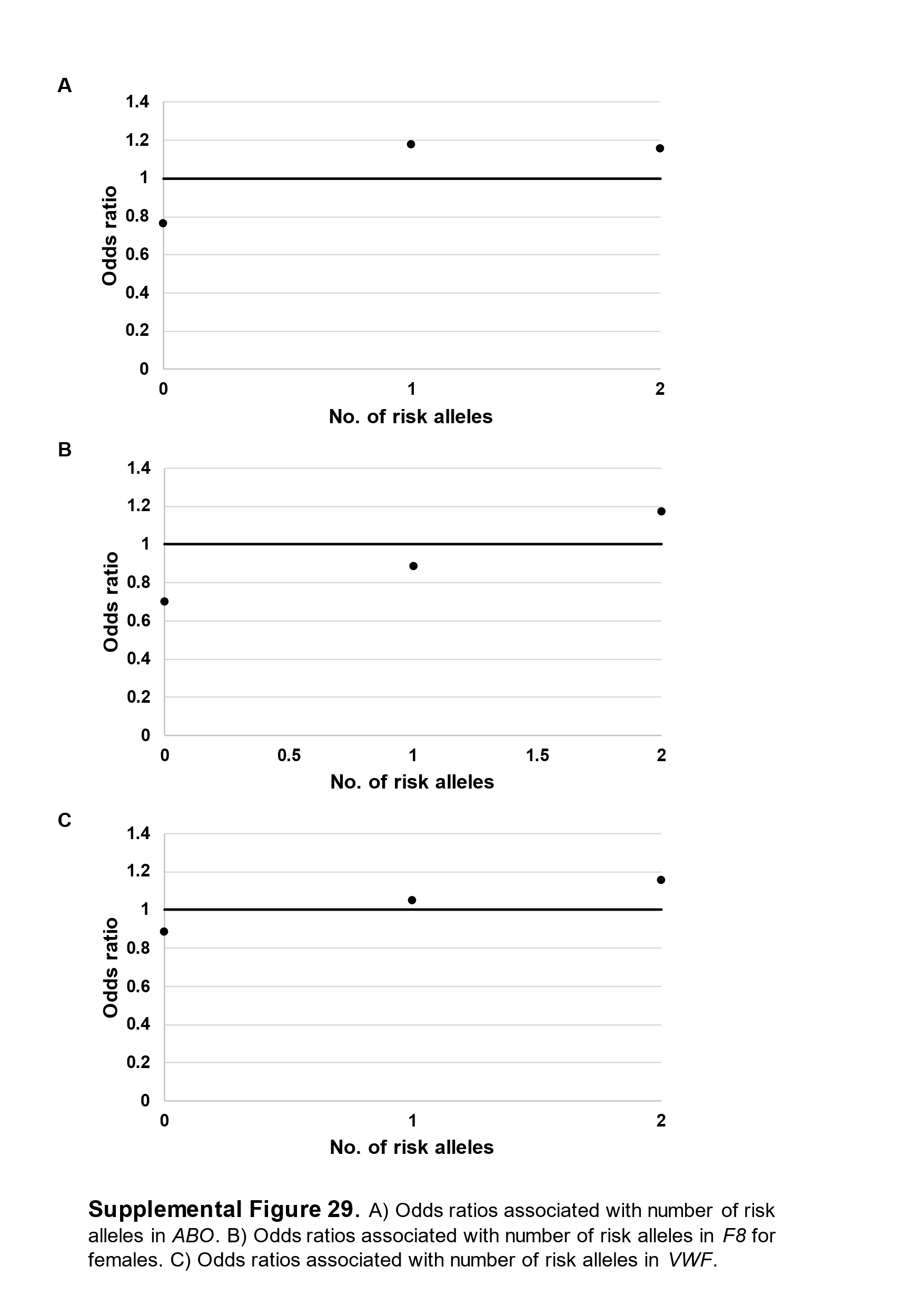


**Supplementary figure 30**


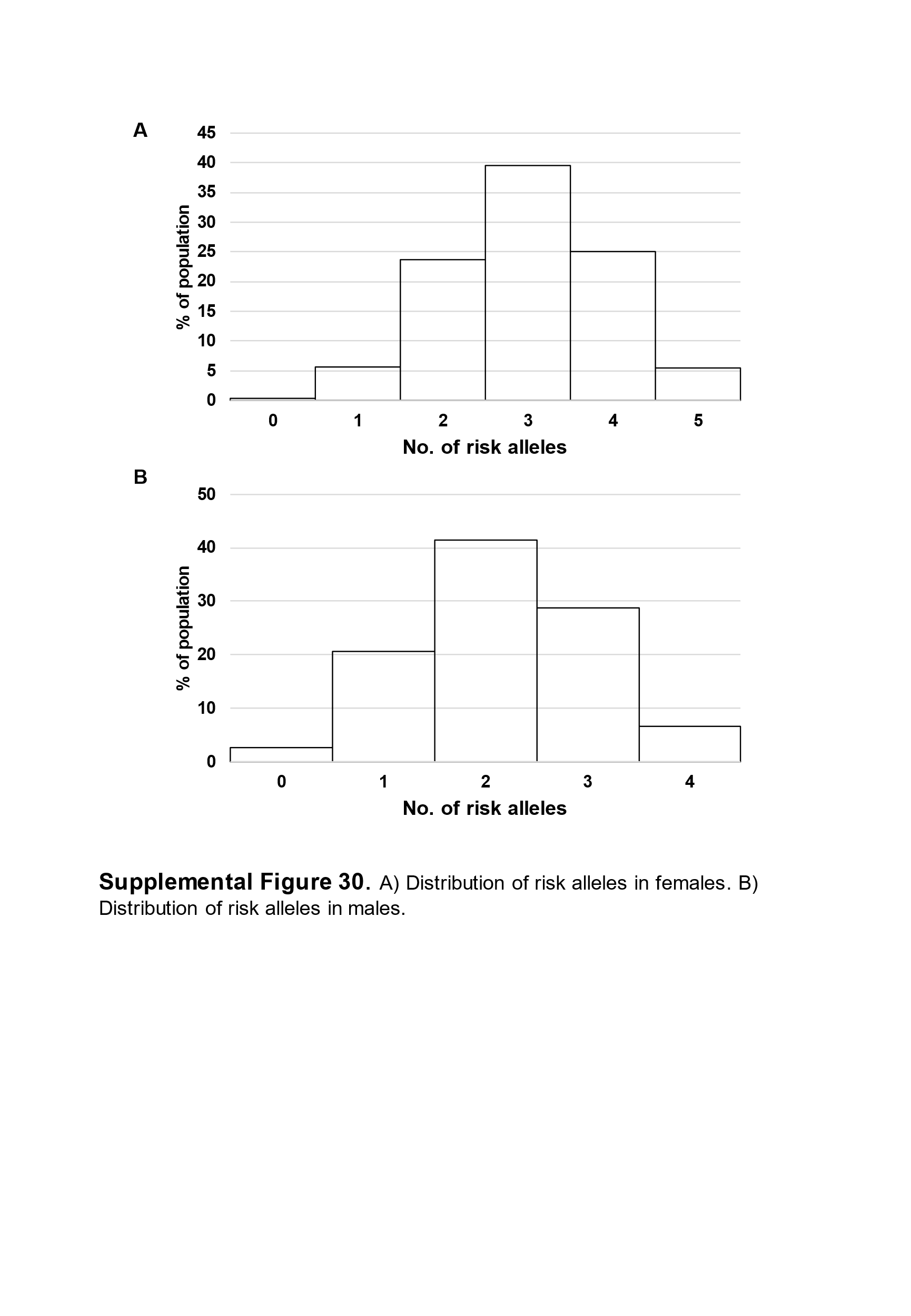


**Supplementary figure 31**


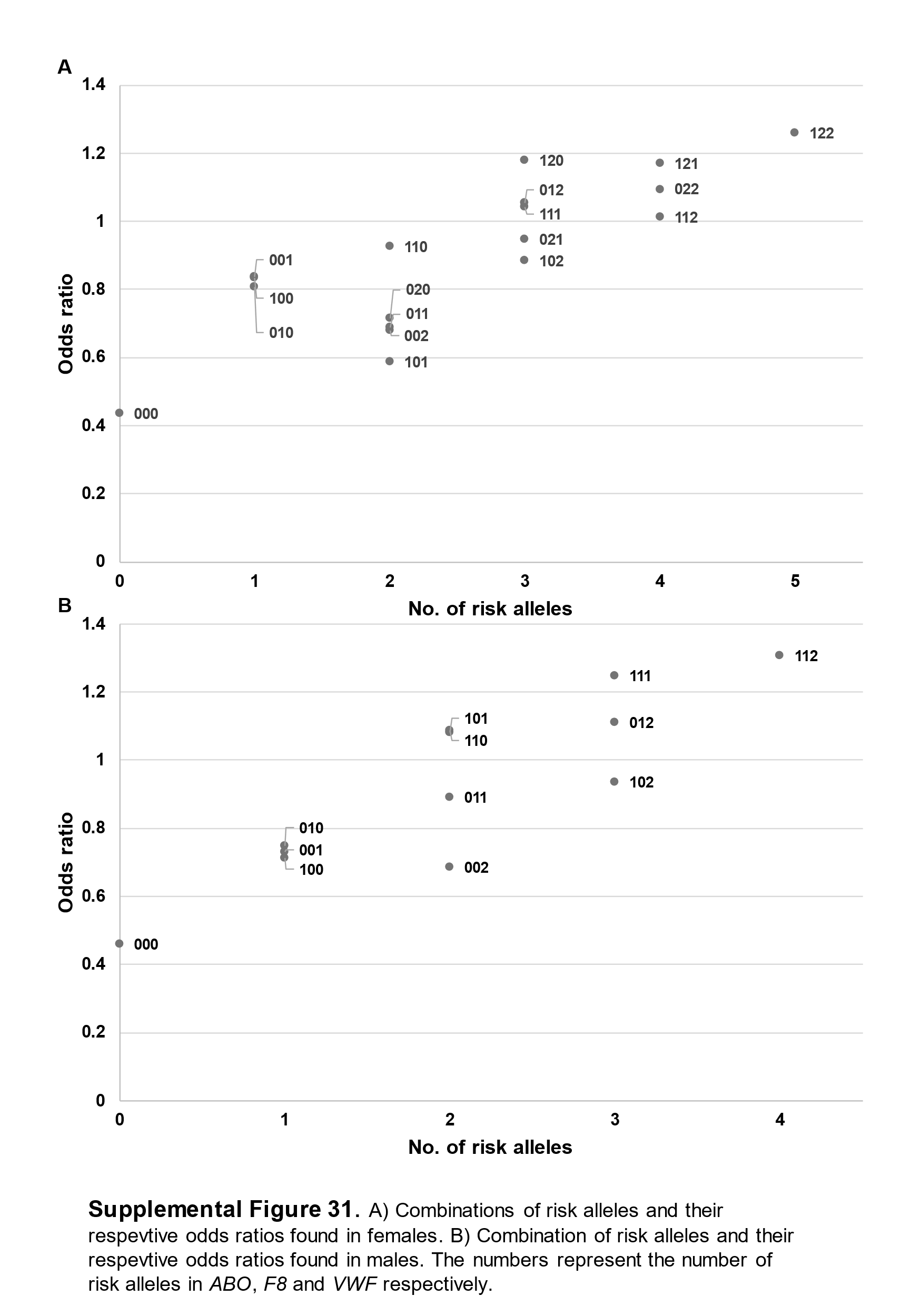


**Supplementary figure 32**

**
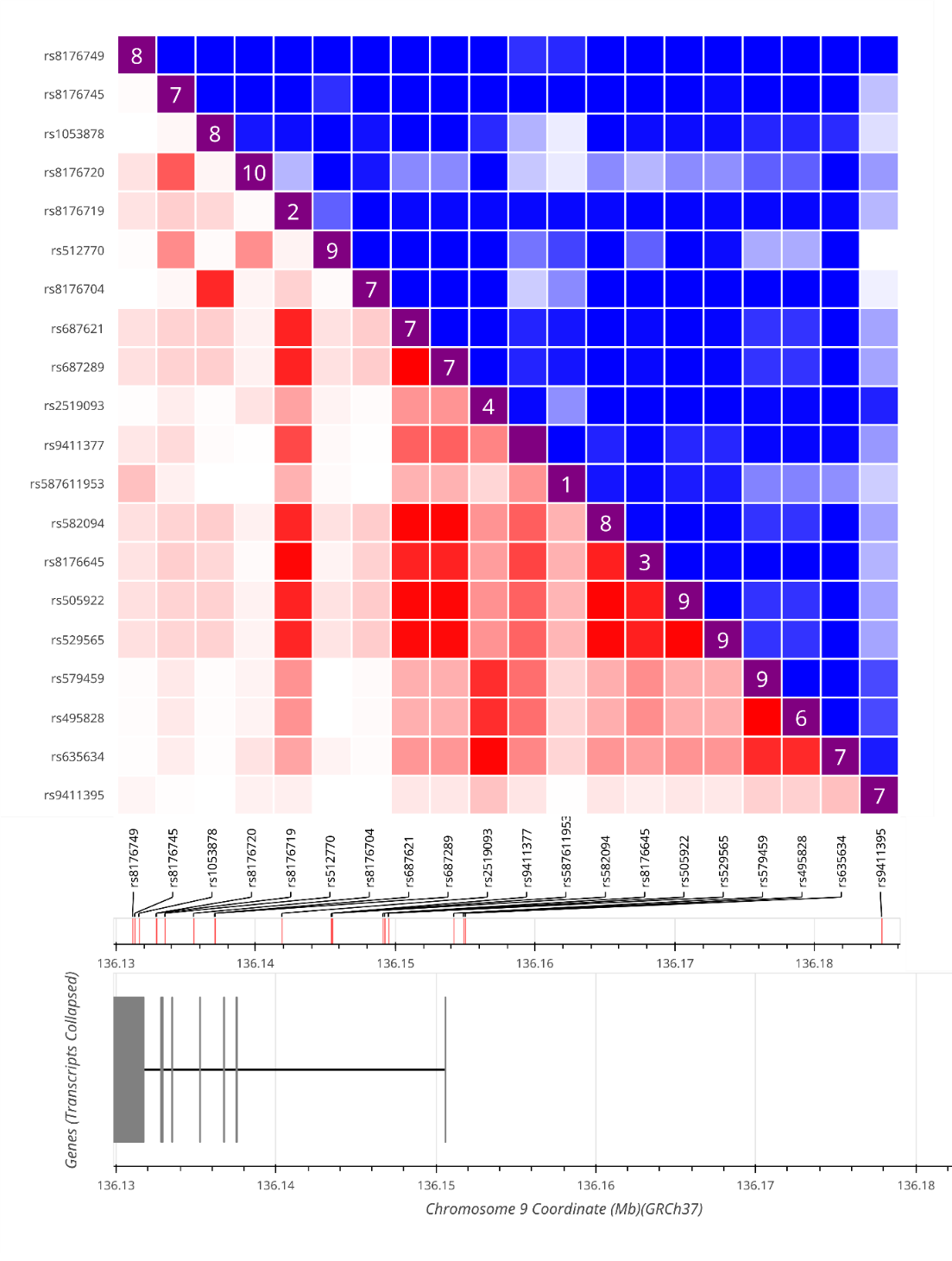
**

**
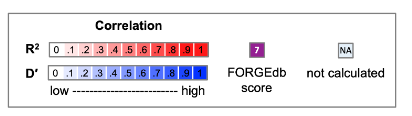
**

**Supplementary figure 32.** Heatmap matrix of pairwise linkage disequilibrium statistic of ABO gene variants created using LDlink [66] among Europeans (EUR) from [Utah Residents from North and West Europe](javascript:void(0);) (CEU) and British in England and Scotland (GBR). The exact r^2^ values are displayed in Supplementary Table 4.

.

**Supplementary figure 33**

**
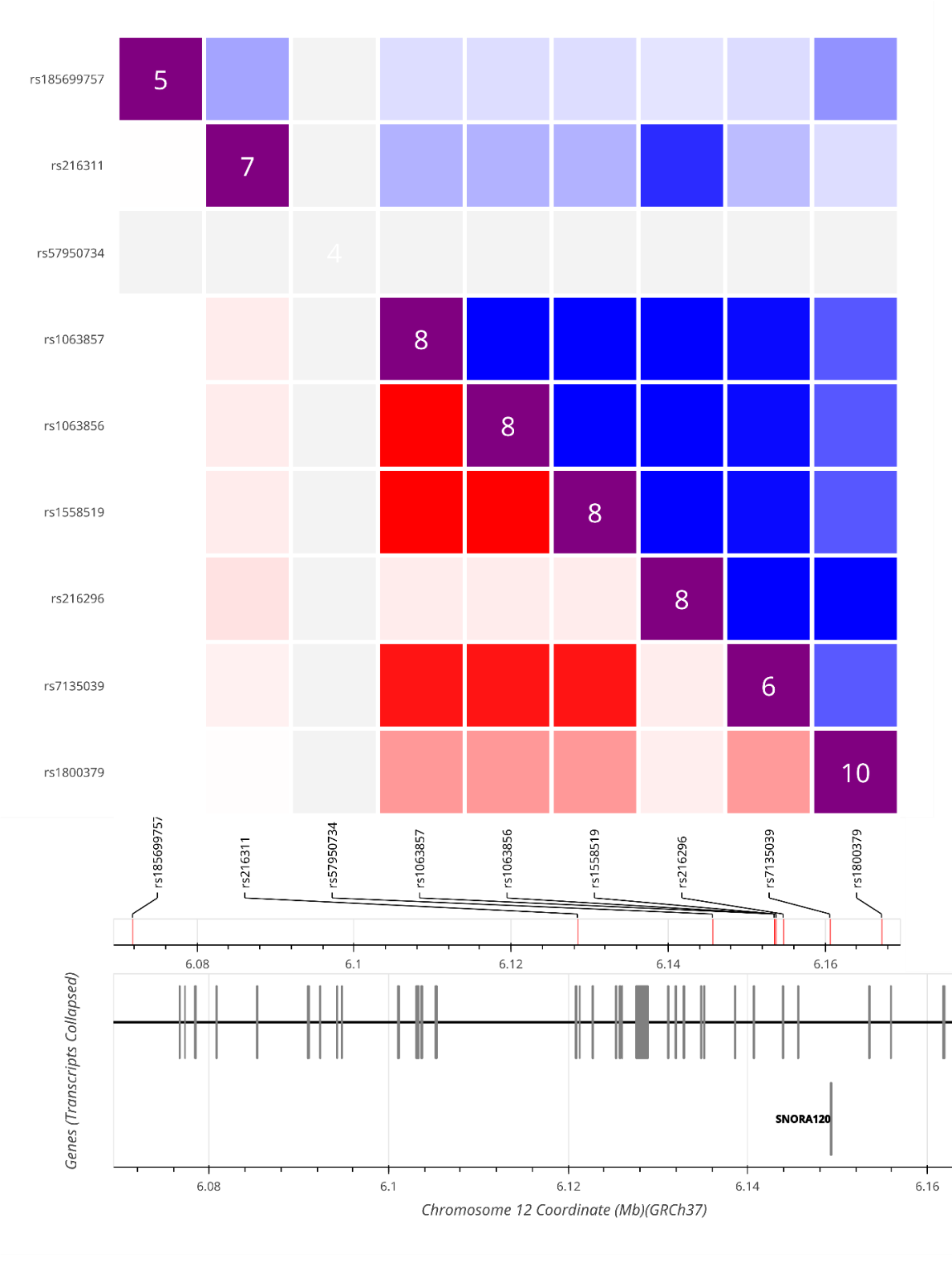
**

**
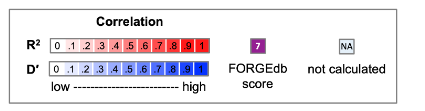
**

**Supplementary figure 33.** Heatmap matrix of pairwise linkage disequilibrium statistic of VWF gene variants created using LDlink [66] among Europeans (EUR) from [Utah Residents from North and West Europe](javascript:void(0);) (CEU) and British in England and Scotland (GBR). The exact r^2^ values are displayed in Supplementary Table 5.

**Supplementary Figure 34.** The predictive model was evaluated on its discriminatory performance and by assessing the agreement between the predicted probability and the observed frequency of outcomes, as described by De Cock et al [68]. This was done by performing the training of the model on 70% of the population (20571 individuals) and tested in the remainder (8816). The calibration of the model showed an intercept of -0.03 (95% confidence interval, -0.10 to 0.03) and slope of 0.95 (95% confidence interval, 0.82 to 1.09).


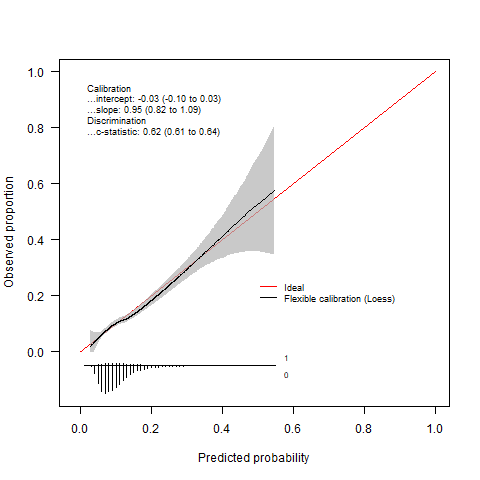

Supplement: Supplementary Material [file mmc1.docx]
